# Corrected Version: IL-12 p40 Monomer is Different from Other IL-12 Family Members to Selectively Inhibit IL-12Rβ1 Internalization and Suppress EAE

**DOI:** 10.33140/jcei.11.03.02

**Published:** 2026-07-13

**Authors:** Susanta Mondal, Madhuchhanda Kundu, Malabendu Jana, Suresh B. Rangasamy, Khushbu K. Modi, Roumen Balabanov, Kalipada Pahan

**Affiliations:** 1 Department of Neurological Sciences, Rush University Medical Center, Chicago, USA; 2 Division of Research and Development, Jesse Brown Veterans Affairs Medical Center, Chicago, USA; 3 Department of Neurology, Northwestern University, Chicago, USA

**Keywords:** IL-12, IL-12p40 monomer, IL-12 p40 homodimer, IL-23, EAE, IL-12Rβ1, IL-12Rβ2, T-helper cells, Regulatory T cells, Inflammatory infiltration, Demyelination, Neuroprotection, multiple sclerosis, EAE

## Abstract

Multiple sclerosis (MS) is the most common human demyelinating disease of the CNS. The IL-12 family of cytokines has four members which are IL-12 (p40:p35), IL-23 (p40:p19), the p40 monomer (p40), and the p40 homodimer (p40_2_). Since all four members contain p40 in different forms, it is important to use specific monoclonal antibody (mAb) to characterize these molecules. Here, by using such mAb, we describe selective loss of p40 in serum of MS patients as compared to healthy controls. Similarly, we also observed decrease in p40 and increase in IL-12, IL-23 and p40_2_ in serum of mice with experimental autoimmune encephalomyelitis (EAE), an animal model of MS, as compared to control mice. Interestingly, weekly supplementation of mouse and human recombinant p40 ameliorated clinical symptoms and disease progression of EAE. On the other hand, IL-12, IL-23 and p402 did not exhibit such inhibitory effect. In addition to EAE, p40 also suppressed collagen-induced arthritis in mice. Using IL-12Rβ1^−/−^, IL-12Rβ2^−/−^ and IL-12Rβ1+/−/IL-12Rβ2^−/−^ mice, we observed that p40 required IL-12Rβ1, but not IL-12Rβ2, to suppress EAE. Interestingly, p40 arrested IL-12-, IL-23- or p40_2_-mediated internalization of IL-12Rβ1, but neither IL-12Rβ2 nor IL-23R, protected regulatory T cells, and suppressed Th1 and Th17 biasness. These studies identify p40 as a new anti-autoimmune cytokine with biological role different from IL-12, IL-23 and p40_2_ in which it attenuates autoimmune signaling via suppression of IL-12Rβ1 internalization, which may be beneficial in patients with MS and other autoimmune disorders.

## 1. Introduction

In the 2020 article by Mondal et al [1], wrong images were provided for the middle two panels (EAE & EAE+p40) of Figure 3G. The middle panel (CIA) of Figure 4D had overlap with another image. The ‘actin’ band of Figure 6D and 6G was same. Similarly, Figure S8K & S8L had overlaps with Figure S9A & S9B. Therefore, the article [1] was retracted. We have corrected these honest mistakes in the current manuscript.

Interleukin-12 (IL-12) plays a critical role in the early inflammatory response to infection and in the generation of T helper type 1 Th-1 cells [2]. IL-12 consists of a heavy chain (p40) and a light chain (p35) linked covalently by disulfide bonds to give rise to the so-called bioactive heterodimeric (p70) molecule [3,4]. The p40 also couples with p19 to generate IL-23 having biological functions that are similar to as well as distinct from IL-12. While similar to IL-12, IL-23 augments the proliferation of Th1 cells to produce more IFN-γ [5,6], contrary to IL-12, IL-23 is known to support the proliferation of memory T cells [5,6]. In addition to forming heterodimers (IL-12 and IL-23), the p40 subunit is also released as p40 monomer (p40) and p40 homodimer (p40_2_) [3,7,8].

Since multiple sclerosis (MS) and its animal model EAE are T cell-driven autoimmune diseases, several studies have reported the involvement of IL-12 in MS and EAE [9–12]. While IL-12 challenge augmented the severity of EAE, antibody to IL-12 inhibited the induction or progression of EAE [13,14]. Similarly, IL-23 also plays a key role in the pathogenesis of MS and p19 (−/−) mice do not develop EAE [15]. On the other hand, the role of p40_2_ and p40 in the disease process of EAE is not known. In general, to investigate the role of a molecule in any disease process, we consider using a knockout mouse model. In this case, however, *p40* (−/−) mice cannot be used because deleting the *p40* gene will knock out IL-12, IL-23, p40_2_, and p40. Therefore, we have generated separate functional blocking monoclonal antibodies (mAb) against p40_2_ and p40 [8]. Recently we have demonstrated that different cancer cells produce greater levels of p40 than p402, IL-12 and IL-23 [16]. Here, we delineate that the level of p40 goes down in MS patients and EAE mice and that weekly treatment with recombinant mouse or human p40 protects mice from EAE. Similarly, p40 also attenuated collagen-induced arthritis in mice. Interestingly, p40 suppressed the internalization of IL-12Rβ1, but neither IL-12Rβ2 nor IL-23R, and attenuated IL-12-, IL-23- and p402-mediated autoimmune signaling pathways. These results delineate a novel anti-autoimmune role of p40, which is different from IL-12, IL-23 and p40_2_.

## 2. Materials and Methods

### 2.1. Reagents:

Bovine myelin basic protein (MBP), L-glutamine and β-mercaptoethanol were obtained from Invitrogen. Fetal bovine serum (FBS) and RPMI 1640 were from Mediatech. Heat-killed *M. tuberculosis* (H37RA) was purchased from Difco Labs. While recombinant mouse p40 monomer (p40) was obtained from BD Bioscience, recombinant human p40 was obtained from R&D systems. Recombinant mouse p40 homodimer (p40_2_) was obtained from R&D systems. Recombinant mouse IL-12 and IL-23 were obtained from eBioscience. Incomplete Freund’s adjuvant (IFA) was obtained from Calbiochem. MOG35–55, solvent blue 38, cresyl violet acetate, and lithium carbonate were purchased from Sigma.

### 2.2. Serum samples of MS patients:

Serum samples of MS patients with active disease were obtained from the MS Clinic of the Rush University Medical Center. These experiments were approved by the Institutional Review Board of the Rush University Medical Center. MS patients are individuals who have been diagnosed with the disease based on the McDonald criteria and were in a state of an acute disease relapse that was independently confirmed by magnetic resonance imaging. Patients were either treatment naïve or off disease-modifying therapy for more than 6 months.

### 2.3. Induction of adoptive transfer EAE in female SJL/J mice by MBP-primed T cells, adoptive transfer EAE in male C57/BL6 mice by MOG35–55-primed Th17 cells, active induction of EAE in female SJL/J mice by PLP139–151, and active induction of EAE in male C57/BL6 mice by MOG35–55:

#### Adoptively-transferred EAE in female SJL/J mice:

Specific pathogen-free female SJL/J mice (5–6 week old) were purchased from Harlan Sprague-Dawley (Indianapolis, IN). EAE was elicited by passive transfer of MBP-reactive T cells as described earlier [17–21]. Donor mice were immunized s.c. with 400 μg bovine MBP and 60 μg *M. tuberculosis* in IFA. Animals were killed 10–12 days post-immunization, and the draining lymph nodes were harvested. Single cell suspensions were treated with RBC lysis buffer (Sigma-Aldrich), washed, and cultured at a concentration of 4–5 × 10^6^ cells/ml in six-well plates in RPMI 1640 supplemented with 10% FBS, 50 μg/ml MBP, 50 μM 2-ME, 2 mM L-glutamine, 100 U/ml penicillin, and 100 μg/ml streptomycin. On day 4, cells were harvested and resuspended in HBSS. A total of 2 × 10^7^ viable cells in a volume of 200 μl was injected into the tail vein of naive mice. Pertussis toxin (150 ng/mouse, Sigma-Aldrich) was injected once via i.p. route on 0-day post-transfer (dpt) of cells. Cells isolated from donor mice immunized with CFA or IFA alone were not viable after 4 days in culture with MBP, and therefore were not transferred. Animal maintenance and experimental protocols were approved by the Rush University Medical Center. Animals were observed daily for clinical symptoms. Experimental animals were scored by a masked investigator, as follows: 0, no clinical disease, 0.5, piloerection, 1, tail weakness, 1.5, tail paralysis, 2, hind limb weakness, 3, hind limb paralysis, 3.5, forelimb weakness, 4, forelimb paralysis, 5, moribund or death.

### 2.4. Adoptive-transfer of EAE in male C57/BL6 mice with MOG-primed Th17 cells:

Male C57BL/6 mice (8–10 wk old) were immunized s.c. with 200 μg bovine MOG35–55 and 60 μg *M. tuberculosis* in IFA. On 10–12 days post-immunization (dpi), lymph node cells (LNC) were isolated to re-stimulate with 10 μg/ml MOG with 2 μg/ml each of plate-bound anti-CD3 and antiCD28, under Th17 polarizing condition (5 ng/ml rhTGFβ + 20 ng/ml rmIL-6 + 20 μg /ml anti-IFNγ) condition. After 2 days, cells were re-plated in the absence of CD3/CD28 but in the presence of 20 ng/ml rmIL-23 for an additional 3 days. Polarization was checked by FACS analysis (Figure S1). Naïve C57/BL6 mice received 2 × 10^7^ viable cells in a volume of 200 μl via tail-vein injection. Pertussis toxin (150 ng/mouse, Sigma- Aldrich) was injected once via i.p. route on 0 dpt.

### 2.5. Induction of relapsing-remitting EAE by PLP139–151 peptide:

EAE was induced in female SJL/J mice (5–6 weeks) by immunization with 100 μg of the PLP139–151 peptide with 60 μg *M. tuberculosis* in IFA. Pertussis toxin (150 ng/mouse) was injected once via i.p. on 0 dpi.

### 2.6. Active induction of EAE by MOG35–55 in male C57/BL6 mice:

C57BL/6 mice (8–10-week-old) were immunized with 100 μg of MOG35–55 as described [18]. Mice also received two doses of pertussis toxin (150 ng/mouse) on 0 and 2 dpi.

### 2.7. Treatment with recombinant p40:

EAE mice were treated with either mouse or human p40 (carrier free) once a week via i.p. injection. Control mice received only PBS. Results were statistically analyzed by the RS/1 multicomparison procedure using a one-way ANOVA and Dunnett’s test for multiple comparisons with a common control group.

### 2.8. Induction of collagen-induced arthritis (CIA):

CIA was induced in male DBA/1J mice (8–9-week-old) as described by us [22]. Briefly, mice were immunized intradermally at the base of the tail with 100 μg of bovine type II collagen emulsified in Incomplete Freund's Adjuvant and M. tuberculosis H37RA. On 21 dpi, mice were boosted with an intraperitoneal injection of 100 μg of bovine type II collagen. Mice were treated p40 (200 ng/mouse/week) i.p. starting from 29 dpi.

### 2.9. Isolation of LNC from p40-treated recipient (EAE) mice:

Female SJL/J mice were induced EAE by adoptive transfer of MBP-primed T cells as described above. From 8 dpt (the onset of acute phase), mice were treated (i.p.) with p40 (200 ng/mouse/week). On 16 dpt, lymph nodes were collected, and LNC were tested for different experiments.

### 2.10. Histological Analysis:

At the peak of the acute phase, mice were anesthetized and perfused with PBS (pH 7.4) and then with 4% (w/v) paraformaldehyde solution in PBS followed by dissection of cerebellum and whole spinal cord from each mouse. The tissues were further fixed and then divided into two halves: one-half was used for histology analysis whereas the other half for myelin staining as described earlier [17–20]. For histological analysis, routine histology was performed to obtain perivascular cuffing and morphological details of spinal cord and cerebellar tissues. Paraformaldehyde-fixed tissues were embedded in paraffin, and serial sections (4 μm) cut. Sections were stained with conventional H&E staining method. Digital images were collected under bright-field setting using an x40 objective. Slides were assessed in a blinded fashion for inflammation by three examiners in different anatomical compartments (meninges and parenchyma). Inflammation was scored using the following scale as described: for meninges and parenchyma: 0, no infiltrating cells, 1, few infiltrating cells, 2, numerous infiltrating cells, and 3, widespread infiltration. For vessels: 0, no cuffed vessel, 1, one or two cuffed vessels per section, 2, three to five cuffed vessels per section and 3, more than five cuffed vessels per section.

### 2.11. Assessment of blood-brain barrier (BBB) and blood-spinal cord barrier (BSB) permeability:

It was performed as described before [18–20]. Briefly, on 14 dpt (acute phase), mice received 200 μl of 20 μM Alexa 680-SE-NIR dye (Invitrogen) via tail vain. After 2 h, mice were scanned in Odyssey (ODY-0854, Licor-Inc) infrared scanner at 700- and 800-nm channels followed by perfusion with 4% paraformaldehyde. Spinal cord and different regions of brain were scanned in Odyssey infrared scanner. The red background came from 800nm filter, whereas the green signal was from Alexa 680 dye at 700 nm channel. The density of the Alexa 680 signal was quantified with the help of Quantity One, version 4.6.2 software using the volume contour tool analysis module.

### 2.12. Staining for Myelin:

Serial longitudinal sections of paraformaldehyde-fixed spinal cords were stained with Luxol fast blue for myelin as described earlier [17–19]. Slides were assessed in a blinded fashion for demyelination by three examiners using the following scale: 0, normal white matter, 1, rare foci, 2, a few areas of demyelination, and 3, large areas of demyelination.

### 2.13. Flow Cytometry:

Single-cell suspensions isolated from mouse spleen, cerebellum or spinal cord were stained with Zombie Aqua^™^ Fixable Viability Kit (Biolegend) according to the manufacturer’s instructions as described by us before [23, 24]. Cells were washed with FACS buffer (ThermoFisher) and stained with CD3-Brilliant Violet 605, CD4-FITC and CD8-APC-Cy7 (Biolegend) for extracellular stains. For intracellular staining, cells were stained according to manufacturer’s instructions using the eBioscience^™^ Foxp3/Transcription Factor Staining Buffer set (ThermoFisher). Cells were then stained with anti-IL-17-APC, anti-IFNγ-PE, anti-GM-CSF-PE-Cy7 (Biolegend), and anti-Foxp3-APC (ThermoFisher). For details on antibodies, please see Table 1. Multicolor flow cytometric analyses were performed using the LSRFortessa analyzer (BD Biosciences) and analyzed using the FlowJo Software (v10).

### 2.14. Semi-quantitative RT-PCR and real-time PCR analyses:

Total RNA was isolated from spinal cord by using RNeasy mini kit (Qiagen) following manufacturer’s protocol. To remove any contaminating genomic DNA, total RNA was digested with DNase. Semi-quantitative RT-PCR was carried out as described earlier [18, 25, 26] using a RT-PCR kit from Clonetech. Briefly, 1 μg of total RNA was reverse transcribed using oligo(dT)12–18 as primer and MMLV reverse transcriptase (Clontech) in a 20 μl reaction mixture. The resulting cDNA was appropriately-diluted, and diluted cDNA was amplified using Titanium Taq DNA polymerase and primers (Table 2). Amplified products were electrophoresed on a 1.8% agarose gels and visualized by ethidium bromide staining.

Real-time PCR was performed using the ABI-Prism7700 sequence detection system (Applied Biosystems) as described earlier [18,25,26]. The mRNA expressions of respective genes were normalized to the level of GAPDH mRNA. Data were processed by the ABI Sequence Detection System 1.6 software and analyzed by ANOVA.

### 2.15. Western blot:

It was performed as described before [27–29]. Briefly, cells were homogenized in RIPA buffer. The supernatant was collected and analyzed for protein concentration via the Bradford method (Bio-Rad). SDS sample buffer was added to protein samples and boiled for 5 min. Denatured samples were electrophoresed on 10 or 12% Bis-Tris SDS polyacrylamide gels in a continuous buffer system, transferred onto a nitrocellulose membrane (Bio-Rad) using the Thermo-Pierce Fast Semi-Dry Blotter. The membrane was then washed for 15 min in TBS plus Tween 20 (TBST) and blocked for 1 h in TBST containing BSA. Next, membranes were incubated at 4°C under shaking conditions with primary antibodies followed by washing of membranes in TBST for 1 h. Membranes were then incubated in secondary antibodies for 1 h at room temperature, washed for 1 more hour, and visualized under the Odyssey Infrared Imaging System (LiCOR Biosciences). Blots were converted to binary, analyzed using ImageJ (NIH), and normalized to the β-actin loading control.

### 2.16. Quantification of p40 and p40_2_ in serum:

Concentrations of p40 and p402 were measured in serum by a sandwich ELISA as described by us [8,19]. While for p40 ELISA, mAb a3–3a was used as coating antibody and mAb a3–7g as detection antibody, for p40_2_ ELISA, mAb a3–1d was employed as coating antibody and mAb d7–12c as detection antibody. During coating, either a3–3a or a3–1d (1 mg/ml) was diluted 1/1000 and added to each well (100 μl per well) of a 96 well ELISA plate. Similarly, during detection, biotinylated a3–7g or d7–12c (1 mg/ml) was diluted 1/1000 and was used as detection antibody.

### 2.17. Statistical Analysis:

Levels of significance for comparison between two groups were determined by one-sided two-sample Mann-Whitney rank-sum test and the Student t test distribution. Analyses were performed by GraphPad Prism 7.02 software. ANOVA were used to compare p40, p40_2_ and IL-12 among different groups. Wherever required, repeated measures One-way ANOVA was employed. Pair-wise comparisons with Bonferroni correction were performed to find out if there was any significant difference detected from ANOVA.

## 3. Results

### Selective decrease in p40 in mice with relapsing-remitting EAE (RR-EAE):

**Figure 1:**
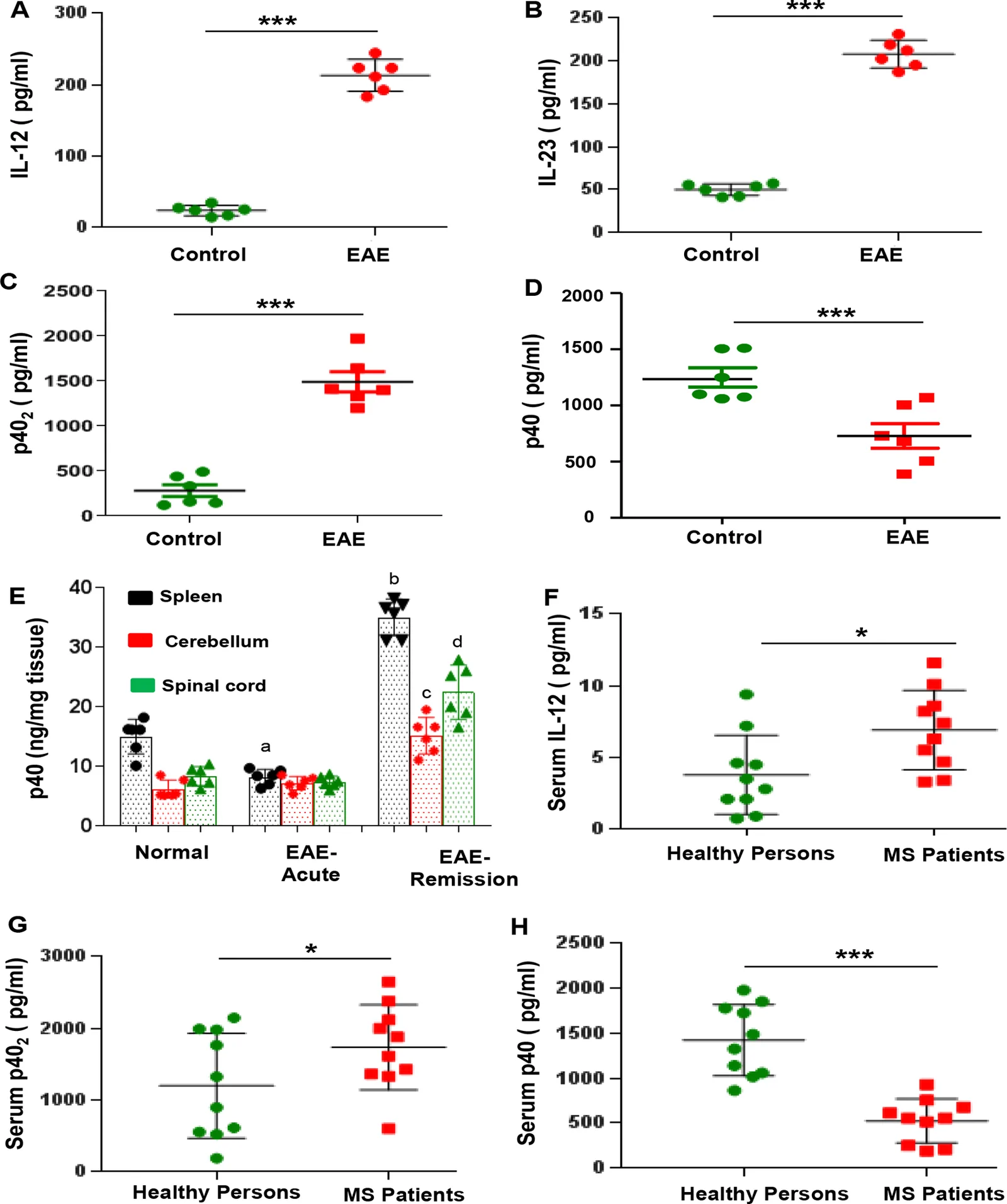
Level of p40 in serum of EAE mice and MS patients. Female SJL/J mice were induced EAE by adoptive transfer of MBP-primed T cells. At the acute phase (14 dpt), levels of IL-12 (A), IL-23 (B), p40 (C), and p40_2_ (D) were measured in serum by sandwich ELISA. Results are mean + SEM of six mice per group. Level of p40 was measured in homogenates of spleen, cerebellum and spinal cord at acute (14 dpt) and remission (22 dpt) phases of EAE (E). Results are mean + SEM of six mice per group. ^*a*^*p* < *0.01* vs control, ^*b*^*p* < *0.001* vs EAE acute phase spleen, ^*c*^*p* < *0.001* vs EAE acute phase cerebellum, ^*d*^*p* < 0.001 vs EAE acute phase spinal cord. Serum of MS patients with active disease (n=10) and age-matched healthy controls (n=10) was analyzed for IL-12 (F), p40_2_ (G) and p40 (H) by sandwich ELISA.

IL-12 p40 is one of the most abundant gene transcripts present in CNS tissues of MS patients and EAE animals [30, 31]. The same p40 gene is responsible for the synthesis of interleukin (IL)-12 p70 (p40: p35), IL-23 (p40: p19), p40 homodimer (p40_2_), and *p40* monomer (p40). Both IL-12 and IL-23 are bioactive cytokines and they play important roles in MS and EAE [5, 11, 32]. Accordingly, we found increased levels of IL-12 and IL-23 in serum of EAE mice at the acute phase of the disease (Figure 1A–B). Consistent to our previous finding [19], the level of p40_2_ was also higher in EAE mice as compared to control mice (Figure 1C). In contrast, the level of p40 was significantly lower in serum of EAE mice at the acute phase compared to control mice (Figure 1D). Accordingly, we also observed loss of p40 in spleen of EAE mice at the acute phase compared to control mice (Figure 1E). In contrast, we did not observe any decrease in p40 in cerebellum and spinal cord at the acute phase of EAE (Figure 1E). However, significant increase in p40 was seen in spleen, cerebellum and spinal cord of EAE mice at the remission phase as compared to acute phase (Figure 1E).

### Levels of p40, p40_2_ and IL-12 in serum of MS patients:

To understand the significance of our finding in MS, we measured levels of p40, p40_2_ and IL-12 in serum of RR-MS patients with acute disease relapse (n=10) and healthy controls (n=10). During blood collection, MS patients were without disease-modifying therapy for more than six months. As reported elsewhere, the level of IL-12 was greater in serum of MS patients than healthy controls (Figure 1F). However, similar to mouse finding, we observed greater [*F*_1,20_ = 6.2108 (>F_*c*_= 4.41), p<0.05 (= 0.022)] levels of p40_2_ (Figure 1G) and lower [*F*_1,20_ = 37.4858 (>F_*c*_= 4.41), p<0.0001 (= 0.00000878)] levels of p40 (Figure 1H) in serum of MS patients than healthy controls.

### Recombinant p40 inhibits disease progression in mice with adoptively-transferred relapsing-remitting (RR)-EAE and chronic EAE:

**Figure 2:**
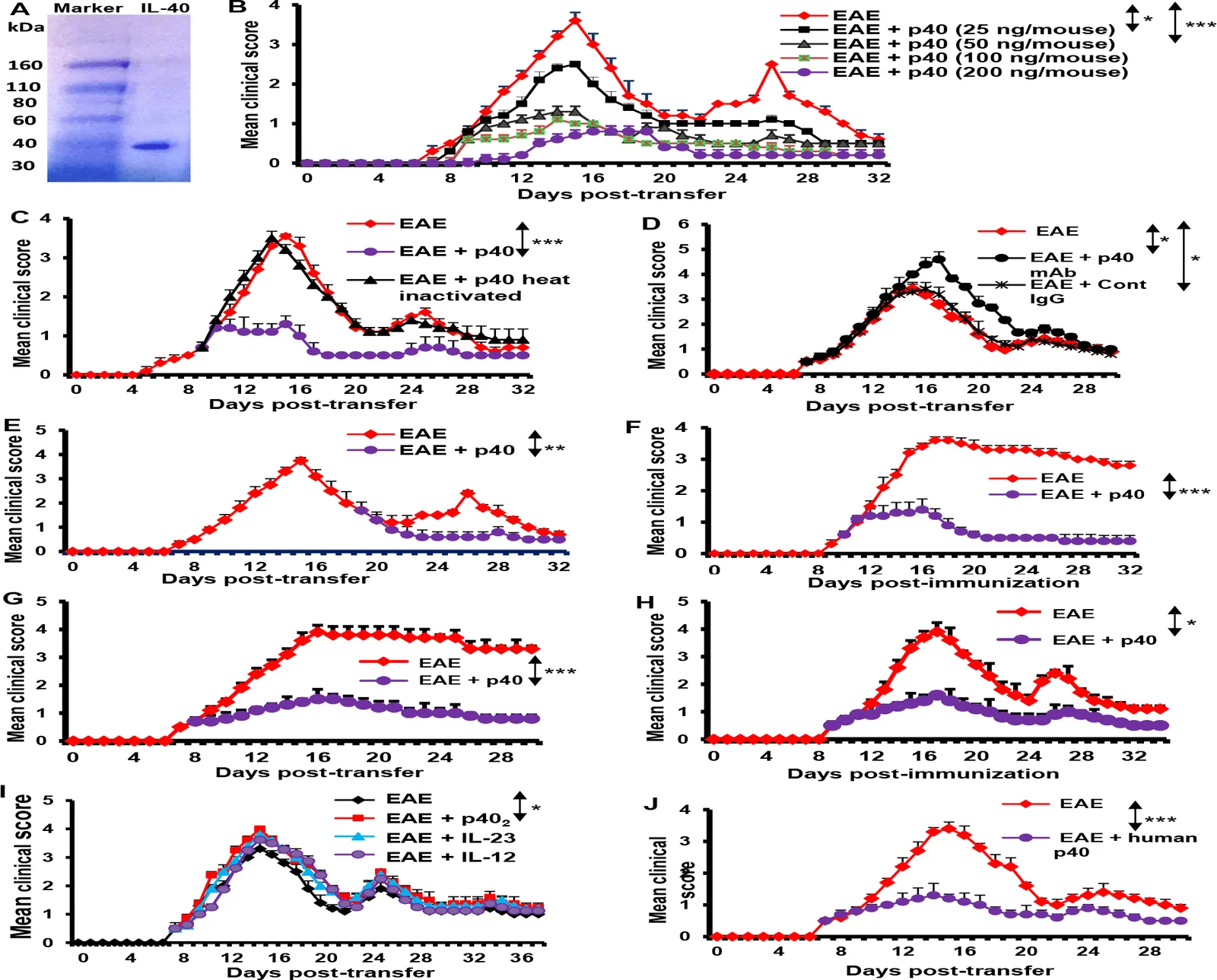
Treatment of EAE by recombinant p40. A) Mouse p40 (BD Bioscience) was run through native PAGE followed by Coomassie blue staining. B) Adoptively-transferred EAE mice were treated with different doses of p40 once a week via i.p. injection starting from 0 dpt. Mice were examined for clinical symptoms for the next 30 days. Data are expressed as the mean ± SEM of six mice per group. *p < 0.05 vs EAE+p40 (25 ng/mouse), ***p < 0.001 vs EAE+p40 (200 ng/mouse). Repeated measures One way ANOVA was calculated with treatment as a single factor and the outcome was summarized as F4,160 = 14.8 (>Fc =4.32). C) Adoptively-transferred EAE mice were treated with recombinant mouse p40 and heat-inactivated p40 (200 ng/mouse) weekly via i.p. injection starting from 8 dpt (the onset of acute phase). Data are expressed as the mean ± SEM of six mice per group. ***p < 0.001 vs EAE+p40. D) Adoptively-transferred EAE mice received one i.p. injection of p40 mAb a3–3a (100 μg/mouse) on 8 dpt. Another group of mice also received same amount of control hamster IgG. Mice were examined for clinical symptoms daily until 30 dpt. Data are expressed as the mean ± SEM of six mice per group. *p < 0.05 vs EAE+p40. E) Adoptively-transferred EAE mice were treated with p40 (200 ng/mouse) weekly starting from 19 dpt (the onset of relapsing phase). Data are expressed as the mean ± SEM of six mice per group. ***p < 0.001 vs EAE+p40. F) MOG-induced active EAE mice were treated with p40 (200 ng/mouse) weekly starting from 10 dpt (the onset of acute phase). Data are expressed as the mean ± SEM of six mice per group. ***p < 0.001 vs EAE+p40. G) Th17 cell-induced active EAE mice were treated with p40 (200 ng/mouse) weekly starting from 8 dpt. Data are expressed as the mean ± SEM of six mice per group. ***p < 0.001 vs EAE+p40. H) PLP139–151-induced chronic relapsing-remitting EAE mice were treated with p40 (200 ng/mouse) weekly starting from 9 dpt. Data are expressed as the mean ± SEM of six mice per group. *p < 0.05 vs EAE+p40. I) Adoptively-transferred EAE mice were treated with recombinant mouse IL-12, IL-23 or p402 (200 ng/mouse) once on 8 dpt. Data are expressed as the mean ± SEM of six mice per group. J) Adoptively-transferred EAE mice were treated with recombinant human p40 (200 ng/mouse) weekly starting from 8 dpt. Data are expressed as the mean ± SEM of six mice per group. ***p < 0.001 vs EAE+p40.

Because the level of p40 decreased in serum of EAE mice and MS patients, we examined if supplementation of mouse recombinant p40 modulates the progression of disease in adoptively-transferred RR-EAE mice. As expected, mouse p40 exhibited ~40 kDa band in non-denaturing PAGE (Figure 2A). Mice were treated weekly with different doses of p40 via i.p. injection from the 0 dpt of MBP-primed T cells. As evident from Figure 2B, p40 dose-dependently inhibited clinical symptoms of EAE and this inhibition was significant with as low as 25 ng p40/mouse [adjusted p = 0.0252 (<0.05) for EAE vs EAE+p40 (25 ng) by Dunnett’s multiple comparison analysis]. However, at higher doses of p40, greater inhibition of EAE [adjusted p = 0.0001 for EAE vs EAE+p40 (50 ng), EAE vs EAE+p40 (100 ng), and EAE vs EAE+p40 (200 ng) by Dunnett’s multiple comparison analysis] was observed (Figure 2B).

Next, mice were treated with p40 from various phases of the disease. In the first group, mice were treated with p40 (200 ng/mouse) from the onset of acute phase (8 dpt). The results in Figure 2C clearly show that the inhibitory effect of p40 on the clinical symptoms was observed within 4 days of treatment (from 12 dpt). There was further marked inhibition on subsequent days of treatment (Figure 2C). However, we did not see any inhibitory effect on clinical symptoms of EAE by heat-killed p40 under the same treatment paradigm (Figure 2C). Moreover, single administration of functional blocking mAb a3–3a against p40, but not control IgG, stimulated clinical symptoms of EAE (Figure 2D), suggesting the specificity of the effect. In the second group, p40 treatment began from the onset of relapsing phase (19 dpt). Figure 2E clearly shows that p40, in this instance, also halted the disease progression. Similarly, p40 treatment from the disease onset also strongly inhibited the clinical symptoms of EAE in MOG35–55-immunized male C57/BL6 mice (Figure 2F), adoptive transfer of Th17 cells (Figure S1A–F) in male C57/BL6 mice (Figure 2G) and PLP139–151-immunized female SJL/J mice (Figure 2H). In contrast, mouse recombinant IL-12, IL-23 and p40_2_ remained unable to suppress the clinical symptoms of RR-EAE in female SJL/J mice (Figure 2I). Since recombinant human p40 is available, we also tested the effect of human p40 on EAE. Similar to recombinant mouse p40, weekly administration of human p40 also suppressed clinical symptoms of adoptively-transferred RR-EAE in female SJL/J mice (Figure 2J).

### Effect of recombinant p40, p40_2_ and IL-12 on the encephalitogenicity of MBP-primed T cells:

Next, we investigated whether p40 was capable of inhibiting the encephalitogenicity of MBP-primed T cells. We found that mice receiving p40-treated MBP-primed T cells displayed significantly reduced clinical symptoms and disease severity (Figure S2A) compared to mice getting only MBP-primed T cells. On the other hand, p40_2_ and IL-12 did not inhibit the encephalitogenicity of MBP-primed T cells. In fact, mice receiving either p402-treated or IL-12-treated MBP-primed T cells exhibited slightly increased disease severity and clinical symptoms (Figure S2A) compared to mice in receipt of only MBP-primed T cells. Next, we investigated whether p40 treatment in donor mice was capable of suppressing the production of encephalitogenic T cells in vivo. Therefore, donor mice were treated with p40, and T cells from these donor mice were re-primed with MBP for 4 d and transferred adoptively to recipient mice. For comparison, groups of donor mice were also treated with p402 and IL-12. We found that mice receiving T cells from p40-treated donor mice displayed less disease severity and clinical symptoms compared to the control EAE group (Figure S2B). These results were specific as we did not observe decrease in clinical symptoms in mice receiving T cells from either p40_2_- or IL-12-treated donor mice (Figure S2B). These results suggest that function of p40 is different from that of either IL-12 or p40_2_ and that p40 is capable of suppressing the encephalitogenicity of MBP-primed T cells.

### The p40, but neither p40_2_ nor IL-12, preserves the integrity of blood-brain barrier (BBB) and blood-spinal cord barrier (BSB) in mice with RR-EAE:

It is known that BBB and BSB break down in a section of the brain and spinal cord, respectively, during active MS and EAE, ultimately allowing different blood molecules and toxins enter into the CNS. We investigated if p40 modulated the integrity of BBB and BSB. As evidenced from Figure S3A (first lane), infrared signals were not visible on areas over the brain and the spinal cord in control HBSS-injected mice. On the other hand, we detected some infrared signals on areas over the brain and the spinal cord of EAE mice (Figure S3A, second column), suggesting possible breakdown of BBB and BSB. The p40_2_ treatment markedly increased the appearance of infrared signals over brain and spinal cord of EAE mice (Figure S3A, compare lane 3 with lane 2). In contrast, p40 treatment suppressed infrared signals over brain and spinal cord (Figure S3A, compare lane 4 with lane 2 and 3). To confirm these results further, the spinal cord and different parts of the brain (frontal cortex, midbrain and cerebellum) were scanned for infrared signals in an Odyssey infrared scanner. Consistent to live mice results, substantial amount of infrared dye was noticeable in CNS tissues of EAE mice as compared to control mice (Figure S3B–F). Again, treatment of EAE mice by p40, but neither p402 nor IL-12, greatly decreased the entry of infrared dye into the spinal cord and different parts of the brain (Figure S3B–F), indicating that biological function of p40 is different from that of either IL-12 or p40_2_.

### The p40, but neither p40_2_ nor IL-12, inhibits the infiltration of mononuclear cells into the CNS of mice with RR-EAE:

**Figure 3:**
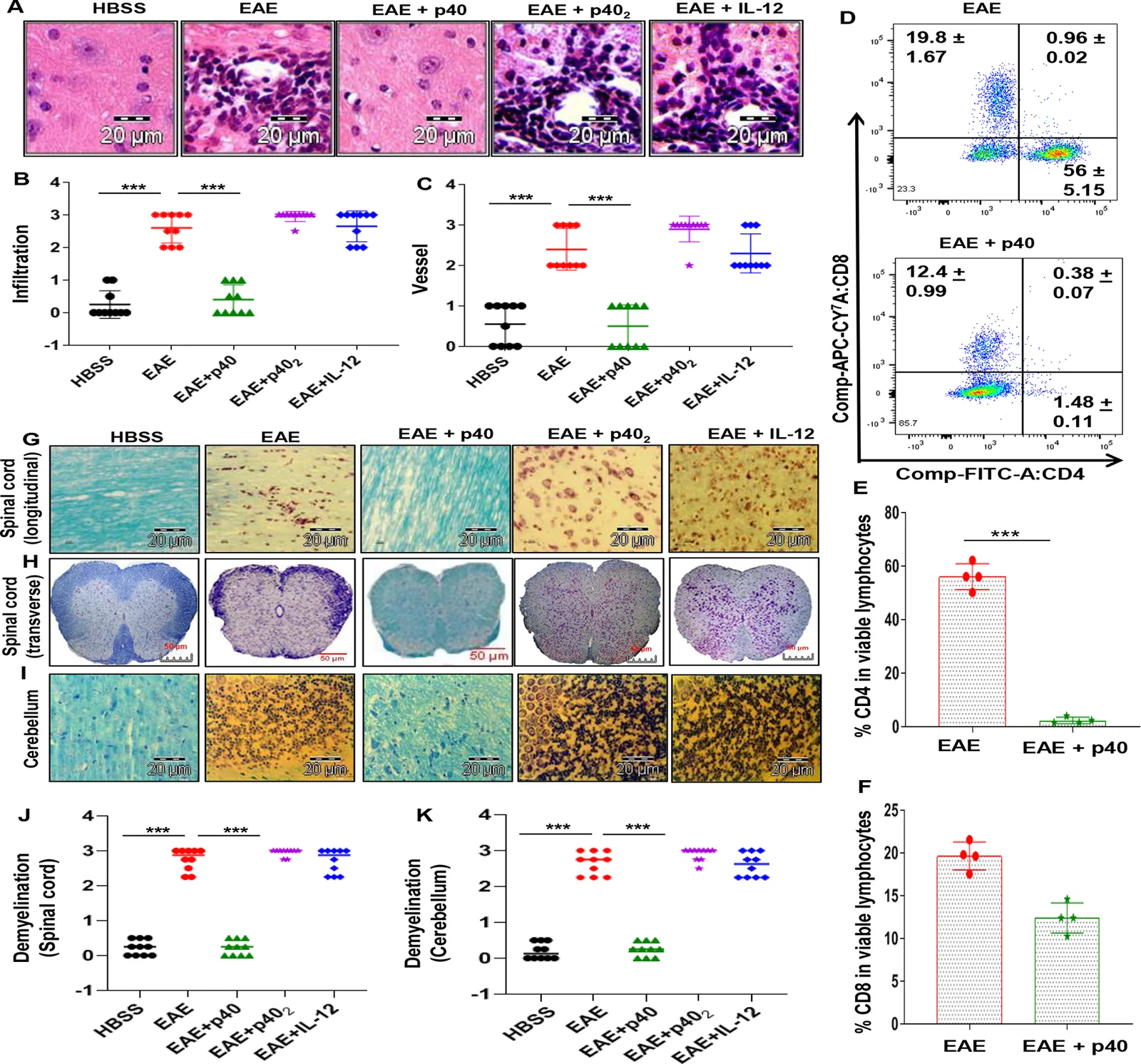
Effect of recombinant p40 on the CNS infiltration of mononuclear cells and demyelination in EAE mice. A) Cerebellar sections isolated from normal, EAE (14 dpt) and p40-, p402- or IL-12-treated EAE (14 dpt receiving these cytokines from 8 dpt) mice were stained with H & E. Digital images were collected under bright field setting using a x 40 objective. Infiltration (B) and cuffed vessel (C) in cerebellar sections were represented quantitatively by using a scale as described in materials and methods. Data are expressed as the mean ± SEM of four mice per group. ap < 0.001 vs control, bp < 0.001 vs EAE. D) Mononuclear cells (MNCs) isolated from the cerebellum of EAE and EAE+p40 mice on 14 dpt were analyzed by FACS in LSRFortessa analyzer (BD Biosciences). Mononuclear cells were gated and percentages of CD4+ (E) and CD8+ (F) T cells in that gate were quantitatively analyzed. Data represent mean ± SEM of 4 mice per group. Longitudinal (G) and transverse (H) sections of spinal cord and coronal sections of cerebellum (I) isolated from normal, RR-EAE (14 dpt) and p40-, p402- or IL-12-treated RR-EAE (14 dpt receiving these cytokines from 8 dpt) mice were stained with Luxol fast blue. Digital images were collected under bright field setting using an x 40 objective. Demyelination in spinal cord (J) and cerebellum (K) was represented quantitatively by using a scale as described in materials and methods. On 8 dpt, mice were treated with 200 ng/mouse p40, p402 or IL-12 via i.p. injection. Data are expressed as the mean ± SEM of four mice per group. ap < 0.001 vs control, bp < 0.001 vs EAE.

It is thought that EAE as well as MS are caused by intrusion of autoreactive T cells and allied mononuclear cells, like macrophages, into the CNS. Accordingly, EAE mice displayed widespread infiltration of inflammatory cells into the cerebellum (Figure 3A). However, treatment of EAE mice with p40, but neither p40_2_ nor IL-12, resulted in decreased intrusion of inflammatory cells into the cerebellum. Quantitation of relative level of inflammation shows that p40, but neither p40_2_ nor IL-12, dramatically reduced infiltration (Figure 3B) and the appearance of cuffed vessels (Figure 3C) in cerebellum of RR-EAE mice. We then analyzed the proportion of CD4^+^ and CD8^+^ T cells from the cerebellum by flow cytometry. While p40 treatment inhibited the entry of both CD4^+^ and CD8^+^ T cells into the cerebellum of EAE mice (Figure 3D–F), the inhibition was very robust for CD4^+^ T cells (Figure 3E).

Infiltration is facilitated by adhesion molecules that are expressed in the endothelium of BBB as well as in glial cells in CNS parenchyma. Therefore, we examined the effect of p40 on the expression of adhesion molecules in cerebellum, spinal cord and optic nerve of EAE mice. Our mRNA analysis data revealed marked expression of ICAM-1 (Figure S4A–C) and P-selectin (Figure S4D–F) in spinal cord (Figure S4A & D), cerebellum (Figure S4B & E) and optic nerve (Figure S4C & F) of EAE mice as compared to control mice. Consistent to the suppression in infiltration, treatment of EAE mice with p40, but neither p402 nor IL-12, resulted in decreased expression of ICAM-1 and P-selectin in spinal cord, cerebellum and otpic nerve as compared to untreated EAE mice (Figure S4A–F). These results again suggest that biological function of P40 is different from that of either IL-12 or p40_2_.

### The p40, but neither p40_2_ nor IL-12, suppresses the expression of pro-inflammatory molecules in CNS tissues of mice with RR-EAE:

We next studied whether p40 was capable of inhibiting the expression of pro-inflammatory molecules in CNS of EAE mice. Marked expression of pro-inflammatory molecules like IL-1μ and iNOS was seen in spinal cord (Figure S4G & J), cerebellum (Figure S4H & K) as well as optic nerve (Figure S4I & L) of EAE mice as compared to control mice. However, treatment of EAE mice with p40, but neither p40_2_ nor IL-12, led to reduction of pro-inflammatory molecule expression in spinal cord, cerebellum and optic nerve of EAE mice (Figure S4G–L).

### The p40, but neither p40_2_ nor IL-12, inhibits demyelination in mice with RR-EAE:

Next, we examined whether p40 protected EAE mice from demyelination. We observed significant decrease in myelin genes like CNPase, MOG, PLP, and MBP in spinal cord (Figure S5A & D), cerebellum (Figure S5B & E) and optic nerve (Figure S5C & F) of EAE mice compared to HBSS-treated control mice. Again, treatment of EAE mice with p40, but neither p40_2_ nor IL-12, led to normalization of myelin gene mRNA expression in spinal cord, cerebellum and optic nerve of EAE mice (Figure S5). To confirm this finding further, we stained spinal cord and cerebellar sections by Luxol fast blue (LFB) for myelin and noticed widespread demyelination zones in the white matter of spinal cord (Figure 3G, H & J) and brain (Figure 3I & K) of EAE mice compared to that of HBSS-treated control mice. However, treatment of RR-EAE mice with p40, but neither p40_2_ nor IL-12, normalized myelin level in both spinal cord (Figure 3G, H & J) and brain (Figure 3I & K). Similarly, p40 treatment also rescued myelin-specific genes and protected myelin in MOG-induced chronic EAE model (Figure S6A–G).

### Weekly p40 treatment suppresses the disease process of collagen-induced arthritis (CIA) in mice:

**Figure 4:**
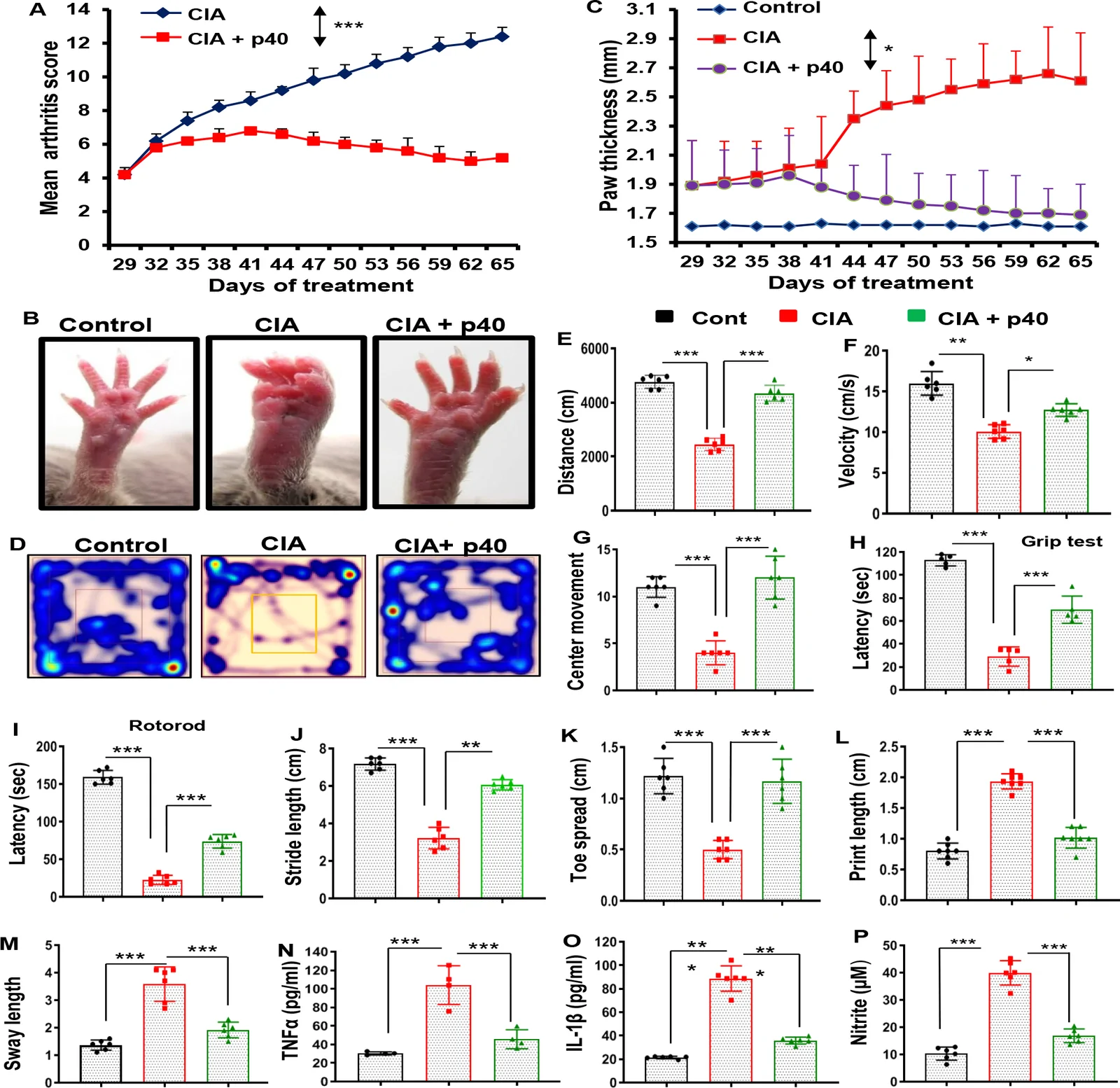
The recombinant p40 protects mice from collagen-induced arthritis (CIA). A) CIA was induced in male DBA/1J mice by bovine type II collagen immunization and from 29 dpi, mice were treated with p40 (200 ng/mouse) weekly via i.p. injection. Mice (n=6 per group in two independent experiments) were scored daily. B) On 60 dpi, images of swollen paws were taken. C) Paw thickness was monitored in six mice per group in two different experiments. Repeated measures One way ANOVA was calculated with treatment as a single factor and the outcome was summarized as F_2,36_ = 50.77 (>Fc =4.68). General motor activities were monitored by Ethovision System (D, heat-map images representing overall motor activities, E, distance travelled, F, velocity, G, center movement), grip strength (H) and rotorod (I). Foot print analysis (J, stride length, K, toe spread, L, print length, M, sway length) was also performed. Levels of TNFα (N), IL-1β (O) and nitrite (P) were also monitored in serum. Six mice (n=6 per group) were used in two independent experiments. *p < 0.05, **p < 0.01 & ***p < 0.001 by two-sample t-tests.

It was important to examine whether the inhibitory effect of p40 was limited to only EAE mice or other autoimmune disease models as well. CIA is a widely-used animal model of rheumatoid arthritis. Similar to EAE mice, p40 also decreased clinical symptoms of CIA in mice (Figure 4A). We also monitored paw swelling and observed marked decrease [adjusted p = 0.0232 (<0.05) by Dunnett’s multiple comparison analysis] in paw thickness upon treatment with p40_2_ (Figure 4B–C). As expected, induction of CIA reduced locomotor activities in mice that are evident by heat-map analysis (Figure 4D), distance traveled (Figure 4E), velocity (Figure 4F), center movement (Figure 4G), grip test latency (Figure 4H), and rotorod (Figure 4I). Footprint analysis (Figure S7) also indicated decrease in stride length (Figure 4J) and toe spread (Figure 4K) and increase in print length (Figure 4L) and sway length (Figure 4M) in CIA mice as compared to normal mice. We also found dragging of toes frequently in CIA mice (Figure S7). However, weekly treatment by p40_2_ improved locomotor activities and normalized footprints in CIA mice (Figure 4D–M & Figure S7).

### The p40 suppresses the internalization of IL-12Rβ1, but neither IL-12Rβ1 nor IL-23R, in T cells: Potential mechanism for the inhibition of IL-12-, IL-23- and p40_2_-mediated signaling:

**Figure 5:**
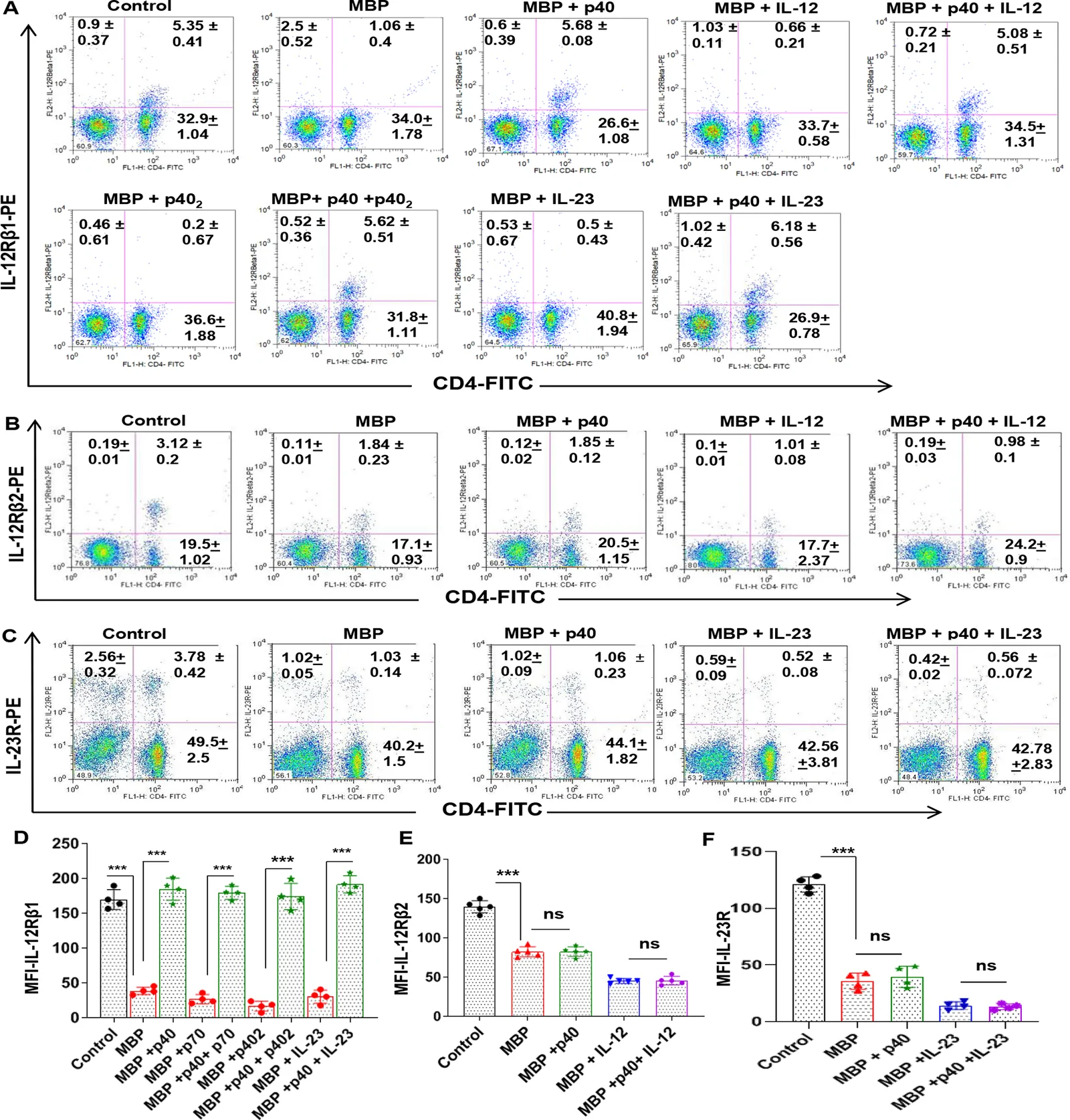
The p40 treatment retains the surface expression of IL-12Rβ1, but neither IL-12Rβ2 nor IL-23R, in MBP-primed T cells. A) Splenocytes isolated from MBP-immunized donor mice were restimulated with MBP in the presence or absence of p40, IL-12, p40+IL-12, p402, p402+p40, IL-23, and IL-23+p40 for 4 h followed by FACS analysis of non-adherent cells in LSRFortessa analyzer (BD Biosciences) for IL-12Rβ1 and CD4. Where cells were treated with the combination of cytokines, p40 was used 30 min prior to p402, IL-12 or IL-23. B) Under similar treatment conditions (p40, IL-12, p40+IL-12), the surface expression of IL-12Rβ2 was monitored by FACS. C) Under similar treatment conditions (p40, IL-23, p40+IL-23), the surface expression of IL-23 was monitored by FACS. The MFI of IL-12Rβ1 (D), IL-12Rβ2 (E) and IL-23R (F) in CD4+ population was calculated by using the CellQuest software. Data are mean ± SD of four different experiments.

To understand the mechanism by which p40 attenuated the disease process of EAE, we investigated the effect of p40 on IL-12, IL-23 and p402 signaling pathways. While IL-12 signals via a heterodimer of IL-12Rβ1 and IL-12Rβ2, IL-23 utilizes a heterodimer of IL-12Rβ1 and IL-23R for functioning [33,34]. We have also demonstrated that p40_2_ participates in the disease process of EAE [19] and that p40_2_ functions via IL-12Rβ1 [35]. After successful binding, these receptors are internalized [36]. Since IL-12Rβ1 is a common receptor subunit for both IL-12 and IL-23, at first, we examined the effect of p40 on the internalization of IL-12Rβ1 in MBP-primed T cells. As evident from Figure 5A & D, normal T cells had much greater surface expression of IL-12Rβ1 than MBP-primed T cells. However, p40 treatment restored the expression of IL-12Rβ1 on the surface of MBP-primed T cells (Figure 5A & D). This result was specific as p402, IL-12 and IL-23 remained unable to restore the surface expression of IL-12Rβ1 in MBP-primed T cells (Figure 5A & D). In fact, p402, IL-12 and IL-23 slightly stimulated the disappearance of IL-12Rβ1 from the surface of MBP-primed T cells (Figure 5A & D). Interestingly, p40 pretreatment reversed the effect of p402, IL-12 and IL-23 and restored the surface expression of IL-12Rβ1 in p40_2_-, IL-12- and IL-23-treated MBP-primed T cells (Figure 5A & D).

To understand the specificity of the effect, we monitored IL-12Rβ2 by FACS analysis. MBP-priming reduced the surface expression of IL-12Rβ2 in T cells and this reduction was more after IL-12 treatment (Figure 5B & E). However, in contrast to the effect on IL-12Rβ1, p40 had no effect on the surface expression of IL-12Rβ2 (Figure 5B & E). To further understand the specificity, we also monitored IL-23R. As expected, we found the reduction of IL-23R on the surface of T cells after MBP-priming, which was further stimulated by IL-23 treatment (Figure 5C & F). However, in contrast to the effect on IL-12Rβ1 and similar to that on IL-12Rβ2, p40 did not modulate the surface expression of IL-23R (Figure 5C & F). Western blot analysis of IL-12Rβ1 in membrane fractions of MBP-primed T cells also confirmed prevention of IL-12Rβ1 internalization by p40 in IL-12- and IL-23-treated and untreated MBP-primed T cells (Figure S8A–F). Pan-cadherin was analyzed to check the purity of the membrane fraction (Figure S8A–F). MBP-priming time-dependently decreased the surface expression of IL-12Rβ1 in T cells (Figure S8A–B) and this decrease was more upon IL-12 (Figure S8C–D) and IL-23 (Figure S8E–F) treatment. However, such loss of IL-12Rβ1 from the membrane was markedly inhibited by p40 (Figure S8A–F).

Again, to understand the specificity, we monitored if p40 modulated the internalization of IL-12Rβ2. At 0 min of MBP re-stimulation, IL-12Rβ2 was present mainly on the membrane (Figure S9A). However, at 2 h or 4 h, IL-12Rβ2 was internalized (Figure S9A). In contrast to IL-12Rβ1, the internalization of IL-12Rβ2 was not inhibited by p40 (Figure S9B). As expected, greater degree of IL-12Rβ2 internalization was observed after IL-12 treatment (Figure S9C). Again, p40 pretreatment was unable to block the internalization of IL-12Rβ2 in IL-12-treated cells (Figure S9D), suggesting that p40 does not have any effect on the internalization of IL-12Rβ2.

**Figure 6:**
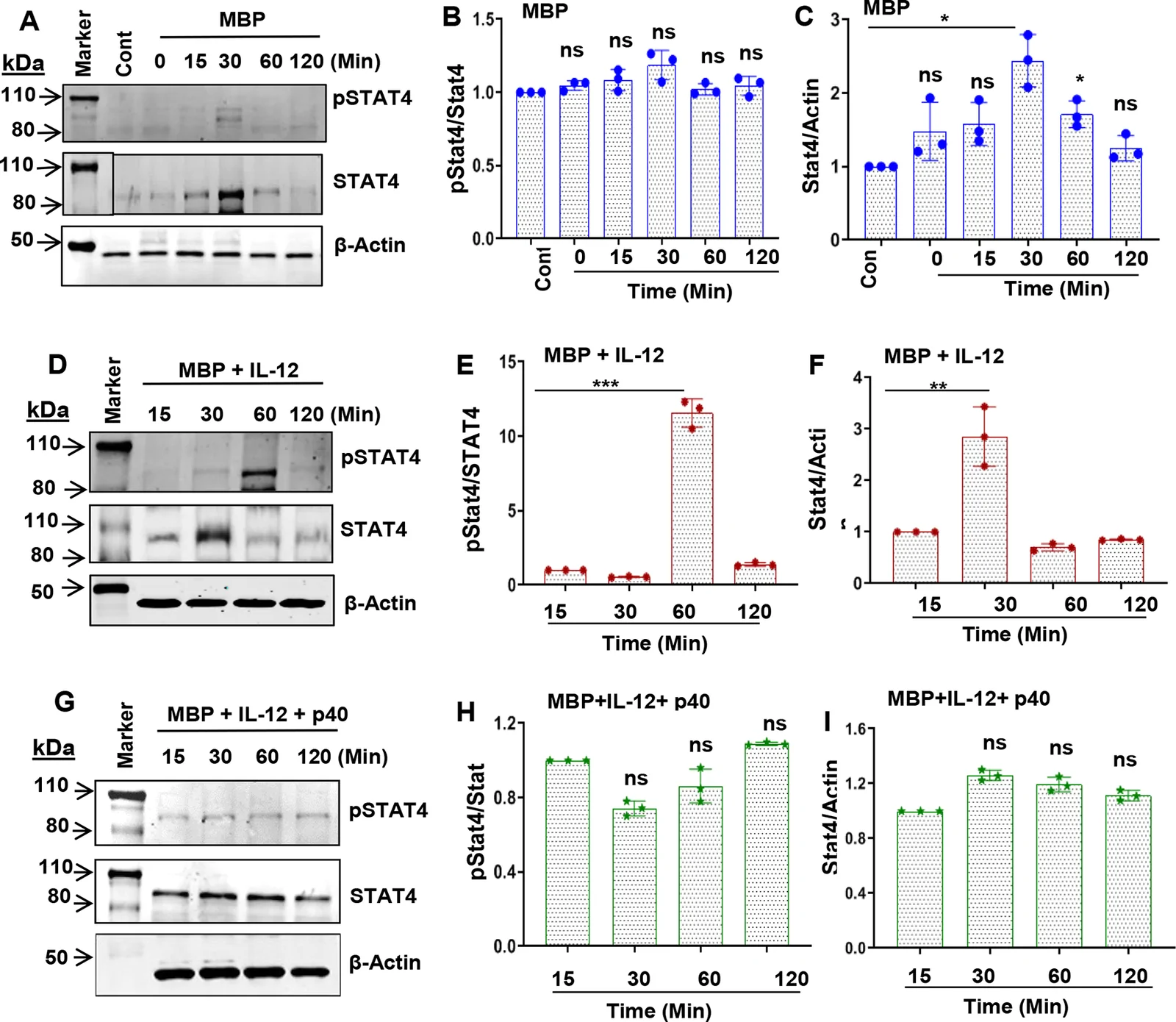
The p40 inhibits the phosphorylation of STAT4 in MBP-primed T cells. Splenocytes isolated from MBP-immunized donor mice were re-stimulated with MBP (A-C) in the presence of IL-12 (D-F) and p40+IL-12 (G-I) for different time periods followed by Western blot of STAT4 and phospho-STAT4 in non-adherent splenocytes. Cells were treated with p40 thirty min before IL-12. Actin was used as a loading control. Bands were scanned and values of pSTAT4/STAT4 (B, MBP, E, MBP+IL-12, H, MBP+IL-12+p40) and STAT4/β-actin (C, MBP, F, MBP+IL-12, I, MBP+IL-12+p40) are presented as relative to control. Data are expressed as the mean ± SD of three independent experiments. *p<0.05, **p<0.01, ***p<0.001, ns, not significant.

To further confirm the inhibition of IL-12Rβ1 internalization by p40, we investigated the effect of p40 downstream signaling events. Since the engagement of IL-12Rβ1 by IL-12 is known to induce the phosphorylation of STAT4 [3], we examined the effect of p40 on the level of phospho-STAT4. Although re-stimulation of MBP-primed splenocytes with MBP led to the increase in total STAT4, we did not observe any significant increase in phospho-STAT4 (Figure 6A–C). However, addition of IL-12 during MBP re-stimulation, markedly increased the phosphorylation of STAT4 (Figure 6D–F), which was strongly inhibited by p40 (Figure 6G–I).

### Enrichment of the Tregs by p40:

**Figure 7:**
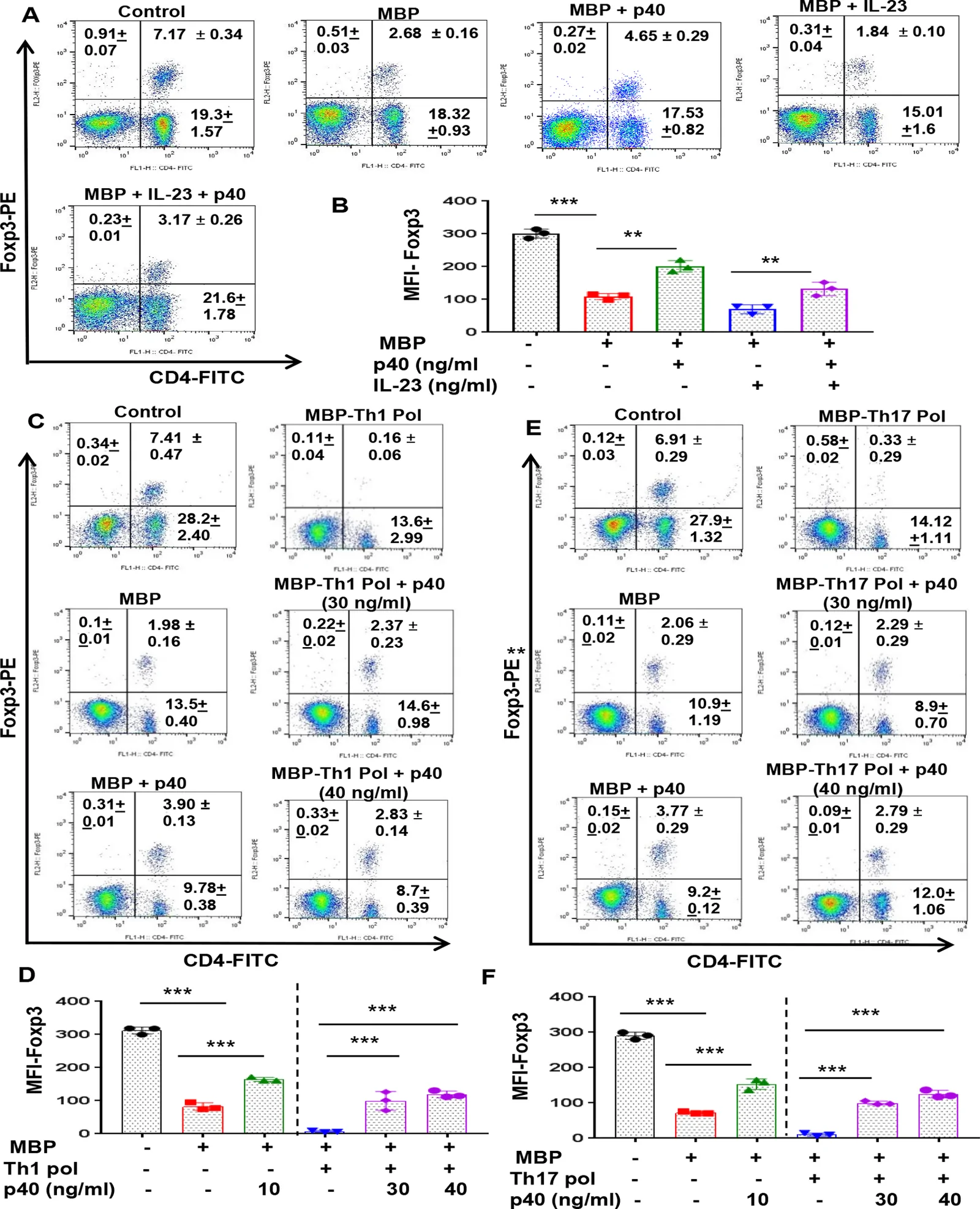
Enrichment of Tregs by p40. Splenocytes isolated from MBP-immunized donor mice were re-stimulated with MBP in the presence or absence of p40 (10 ng/ml), IL-23 (10 ng/ml) and p40+IL-23 for 48 h followed by FACS analysis of non-adherent cells in LSRFortessa analyzer (BD Biosciences) for CD4 and Foxp3 (A). The MFI of Foxp3 (B) in CD4+ population was calculated by using the CellQuest software. Results are mean + SD of three different experiments. **p < 0.01 & ***p < 0.001 by two-sample t-tests. Splenocytes isolated from MBP-immunized donor mice were treated with p40 (30 ng/ml) followed by re-stimulation with MBP in the presence of either Th1 (C-D) or Th17 (E-F) polarization as mentioned under methods section. After 48 h, cells were analyzed by FACS for CD4 and Foxp3 (C, Th1 polarization, E, Th17 polarization). The MFI of Foxp3 (D, Th1 polarization, F, Th17 polarization) in CD4+ population was calculated by using the CellQuest software. Results are mean + SD of three different experiments. ***p < 0.001 by two-sample t-tests.

Until now, Tregs are probably the most important immunomodulatory subtype of T lymphocytes [37,38]. Therefore, in order to comprehend the consequence of p40-mediated inhibition of IL-12Rβ1 signaling, at first, we examined the effect of p40 on the status of Tregs. It has been reported that Tregs become both numerically and functionally imperfect during autoimmune insults [39,40]. Foxp3 is a prototype marker of Tregs and as expected, MBP-priming led to marked decrease in CD4^+^Foxp3^+^ T cells (Figure 7A–B). However, p40 treatment enriched CD4+Foxp3+ T cells in MBP-primed splenocytes (Figure 7A–B). To further endorse this observation, we studied the effect of p40 on the differentiation into Tregs under Th1- or Th17-favoring conditions. MBP-primed splenocytes displayed greater loss of CD4^+^Foxp3^+^ T cells under Th1-polarizing condition than non-polarizing condition (Figure 7C–D). On the other hand, p40 treatment markedly protected CD4^+^Foxp3^+^ T cells under Th1-favoring conditions (Figure 7C–D). However, higher concentrations of p40 were needed to protect CD4^+^Foxp3^+^ T cells under Th1 polarizing condition (Figure 7C–D). Similarly, MBP-primed splenocytes also had very few CD4^+^Foxp3^+^ T cells under Th17-polarizing condition as compared to non-polarizing condition (Figure 7E–F), which was increased by p40 treatment (Figure 7E–F).

Next, we monitored the status of Tregs *in vivo* in EAE mice. EAE mice receiving p40 from 8 dpt were sacrificed on 16 dpt followed by analysis of Tregs in splenocytes and the CNS. As evident from FACS dot plot (Figure S10A) and mean fluorescence intensity (MFI) (Figure S10B), there was a significant reduction in CD4^+^Foxp3^+^ population of T cells in EAE splenocytes as compared to normal splenocytes, which was increased by p40 treatment. Similarly, p40 treatment also upregulated the frequency of CD4^+^Foxp3^+^ T cells in the mononuclear cells extracted from the cerebellum of EAE mice (Figure S10C–H). Although IL-10 is a Th2 cytokines, Tregs also have the ability to produce IL-10. Accordingly, we also observed increase in IL-10 in cerebellum of p40-treated EAE mice as compared to untreated EAE mice (Figure S10I).

### Suppression of Th1 and Th17 responses by p40 treatment:

Next, we investigated the effect of p40 on Th1 and Th17 responses. While MBP-priming increased the level of CD4+IFNγ+ T cells (Figure S11A & C) and the production of IFNγ (Figure S11E) in splenocytes, p40 treatment markedly suppressed MBP-induced upregulation of CD4+IFNγ+ T cell population and IFNγ. Similarly, p40 also inhibited IL-12-mediated upregulation of CD4+IFNγ+ T cells (Figure S11A & C) and IFNγ production (Figure S11E) from MBP-primed splenocytes. As expected, MBP-priming also increased the level of CD4+IL-17+ T cells (Figure S11B & D) and the production of IL-17 (Figure S11F) in splenocytes, which was further stimulated by IL-23. However, p40 markedly suppressed the level of CD4+IL-17+ T cells (Figure S11B & D) and the production of IL-17 (Figure S11F) in MBP-primed and IL-23-treated MBP-primed splenocytes. To further confirm these observations, we examined the effect of p40 on the differentiation of MBP-primed T cells into Th1 and Th17 cell types under Th1- or Th17-polarizing conditions. MBP-primed T cells produced higher level of IFN-γ under Th1-polarizing condition than non-polarizing condition (Figures. S11G & S12A–B). However, p40 markedly inhibited the ability of MBP-primed T cells to induce the production of IFN-γ under Th1-favoring conditions (Figures. S11G & S12A–B). Similarly, MBP-primed T cells expressed greater level of IL-17 under Th17-polarizing condition than non-polarizing condition, which was inhibited by p40 treatment (Figures. S11H & S13A–B). Since results described above deals with antigen-primed T cells, next, pure naïve CD4+ T cells were primed in Th1 or Th17 polarizing conditions in the presence or absence of p40 followed by monitoring both IFNγ and IL-17 at the same time by FACS. As expected, Th1 polarization markedly increased IFNγ, but not IL-17, and Th17 polarization strikingly augmented IL-17, but not IFNγ (Figure S14A–D). However, p40 treatment strongly inhibited IFNγ under Th1 polarizing condition and IL-17 during Th17 polarization (Figure S14A–D). It has been shown that GM-CSF plays an important role in the development of autoimmune CNS inflammation and that the expression of GM-CSF in CD4+ and CD8+ T cells is increased in MS patients [41,42]. Interestingly, p40 strongly inhibited the level of GM-CSF in Th1 cells (Figure S15A–D).

Next, we examined if p40 exhibited similar effects on Th1 and Th17 cells in vivo in the CNS of EAE mice. As evident from Figure S16, p40 treatment of RR-EAE mice led to the inhibition of CD4+IFNγ+ and CD4+IL-17+ T cells in vivo in cerebellum (Figure S16A, C & D) and spinal cord (Figure S16B, E & F). These results indicate that p40 treatment is also capable of suppressing Th1 and Th17 responses in vivo in the CNS of EAE mice.

### The p40 requires IL-12Rβ1 (Rβ1), but not IL-12Rβ2 (Rβ2), to suppress EAE:

**Figure 8:**
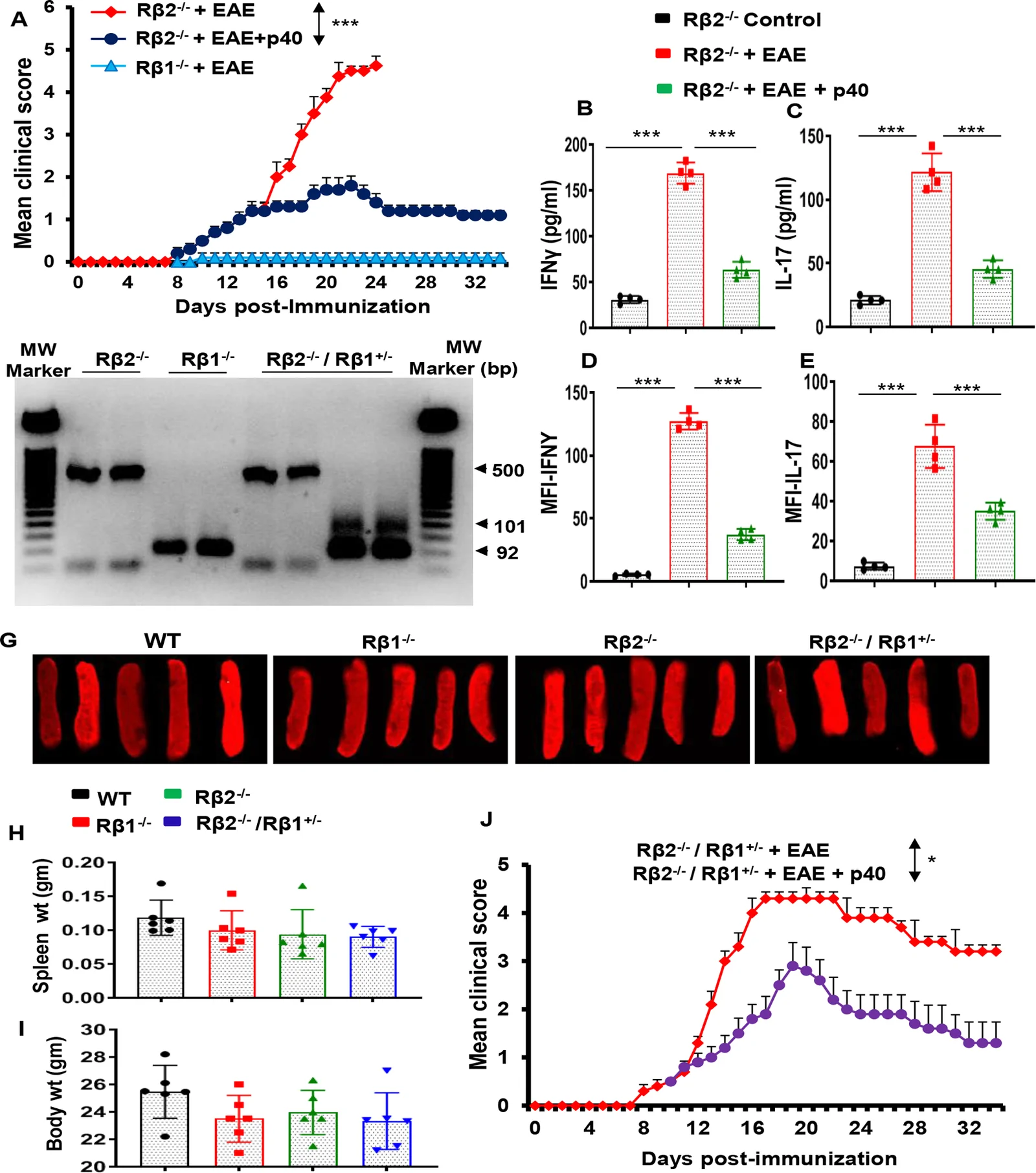
Involvement of IL-12Rβ1 and/or IL-12Rβ2 in p40-mediated protection of EAE. A) EAE was induced in IL-12Rβ1−/− (Rβ1−/−) and IL-12Rβ2−/− (Rβ2−/−) mice by MOG immunization. We did not observe EAE symptoms in IL-12Rβ1−/− mice. From 8 day post-immunization, IL-12Rβ2−/− mice were treated with p40 (200 ng/mouse) once a week via i.p. injection. Mice were examined for clinical symptoms until 34 dpi. Data are expressed as the mean ± SEM of six mice per group. ***p < 0.001 vs EAE+p40. On 20 dpi, levels of IFNγ (B) and IL-17 (C) were monitored in serum by ELISA. Results are mean + SEM of four mice per group. Splenocytes were analyzed by FACS in LSRFortessa analyzer (BD Biosciences) for CD4 & IFNγ and CD4 & IL-17. The MFI of IFNγ (D) and IL-17 (E) in CD4+ population was calculated by using the CellQuest software. ***p < 0.001 by two-sample t-tests. Rβ1−/− mice were bred with Rβ2−/− mice to generate Rβ1+/−/Rβ2−/− mice. Genotyping data are presented (F). Whole spleens are shown for all different groups (G). There were no significant differences in spleen weight (H) and total body weight (I) among different groups of mice (8 weeks old). J) EAE was induced in Rβ1+/−/Rβ2−/− mice by MOG immunization followed by treatment with p40 (200 ng/mouse) once a week via i.p. injection from 8 dpi. Mice were scored until 34 dpi. Data are expressed as the mean ± SEM of six mice per group. *p < 0.05 vs p40 treatment.

Since p40 inhibited the internalization of Rβ1, we investigated the role of Rβ1 and Rβ2 in p40-mediated suppression of EAE. At first, we induced EAE in Rβ1−/− and Rβ2−/− mice. As reported earlier [43], MOG immunization did not induce EAE symptoms in Rβ1−/− mice, but led to severe EAE symptoms in Rβ2−/− mice (Figure 8A). In fact, all MOG-immunized mice in the in Rβ2−/− group went to the moribund stage within 24 dpi (Figure 8A). However, weekly p40 treatment significantly reduced clinical symptoms of EAE in Rβ2−/− mice (Figure 8A), suggesting that p40 requires Rβ1, but not Rβ2, for suppressing EAE. Since Th1 and Th17 cells play an important role in the pathogenesis if EAE, we also monitored the status of these cells in Rβ2−/− EAE mice. Induction of EAE in Rβ2−/− mice led to marked increase in serum levels of IFNγ (Figure 8B) and IL-17 (Figure 8C). However, p40 treatment markedly inhibited the levels of IFNγ and IL-17 in serum of Rβ2−/− EAE mice (Figure 8B–C). Similarly, p40 treatment also suppressed the levels of CD4+IFNγ+ (Figure S17A & Figure 8D) and CD4+IL-17+ (Figure S17B & Figure 8E) T cells in the spleen of Rβ2−/− EAE mice, indicating that p40 inhibits Th1 and Th17 responses via Rβ1, but not Rβ2. Since Rβ1−/− mice are resistant to EAE, to further prove the role of Rβ1 and Rβ2 in p40-mediated suppression of EAE, we crossed Rβ1−/− and Rβ2−/− mice to generate Rβ1+/−/Rβ2−/− mice (Figure 8F), where Rβ1 was partially present. Two-month-old WT, Rβ1−/−, Rβ2−/−, and Rβ1+/−/Rβ2−/− mice did not differ significantly with respect to either wet spleen weight (Figure 8G–H) or gross body weight (Figure 8I). We also did not notice any overt phenotypic differences, including diet, fecal boli, social interaction, and agitation across genotypes at this age. However, upon induction of EAE, we found that the severity of EAE is less in Rβ1+/−/Rβ2−/− mice (Figure 8J) as compared to Rβ2−/− mice (Figure 8A). Although p40 treatment inhibited clinical symptoms of EAE in Rβ1+/−/Rβ2−/− mice, the inhibition was much stronger in Rβ2−/− mice (Figure 8A) than that observed in Rβ1+/−/Rβ2−/− mice (Figure 8J), again suggesting that p40 suppresses the disease process of EAE via Rβ1, but not Rβ2. In Rβ1+/−/Rβ2−/− EAE mice, p40 treatment also led to upregulation of Tregs (Figure S18A & D) and suppression of Th1 (Figure S18B & E) and Th17 (Figure S18C & F) cells. However, p40 was less efficient in Rβ1+/−/Rβ2−/− EAE mice than either WT EAE mice or Rβ2−/− EAE mice in suppressing Th1 and Th17 cells.

## 4. Discussion

MS is the most common autoimmune demyelinating disease of the CNS. Since IL-12 is the most important cytokine in terms of cell-mediated immunity [44], this molecule is considered as an essential component of autoimmunity [3,10,15]. IL-12 family of molecules has four different members including p40 monomer (p40), p40 homodimer (p402), IL-12 (p40:p35), and IL-23 (p40:p19) [3,5]. In the current era of science, where heterodimers rule, only IL-23 and IL-12 were thought to be biologically active. Accordingly, p40 and p40_2_ were considered as inactive members of the IL-12 family [3]. In contrast, we have established the proinflammatory property of p402 [35,45,46] and described that biological activities of p402 are different from that of IL-12 and IL-23 [47,48]. Furthermore, after generating separate functional blocking monoclonal antibodies (mAb) and ELISA against each of mouse p40_2_ and p40 [8], we have demonstrated that neutralization of p40_2_ by specific mAb protects mice from EAE [19]. Recently we have seen that level of p40 is much higher in serum of prostate cancer patients as compared to healthy controls and that neutralization of p40 leads to shrinkage of prostate tumors in mice [16].

Here, we demonstrate that the level of p40 goes down in MS patients and EAE animals and that treatment with recombinant mouse or human p40 protects mice from EAE. The p40 also inhibited the disease process of CIA, an animal model of rheumatoid arthritis. This is the first report demonstrating an anti-autoimmune role of p40 that is different from other members of the IL-12 family. These results also indicate a possible therapeutic prospect of recombinant p40 in MS. Recently a phase II clinical trial of ustekinumab, an anti-IL-12/23p40 antibody, found no clinical or radiologic improvement in any MS patient treatment group compared with placebo controls [49]. It is expected because ustekinumab neutralizes p40 present in IL-12, IL-23, p40_2_, and p40. Although IL-12, IL-23 and possibly p402 are involved in the disease process of MS, according to our results, p40 is protective for EAE and most likely for MS as well. Therefore, by neutralizing both autoimmune (IL-12, IL-23 and p40_2_) and anti-autoimmune (p40) components at the same time, ustekinumab should not be protective in MS patients.

Regulatory T cells (Tregs), viewed as the master controller of immune responses, play vital roles in MS and EAE [37,38,50,51]. Foxp3 is considered as a prototype marker of Tregs and several studies demonstrate the decrease in Foxp3 and reduction of Foxp3^+^ T cells in relapsing-remitting MS patients compared with those in control subjects [39,40]. Therefore, upregulation and/or maintenance of Tregs may be beneficial for MS. Here, we demonstrate that p40 treatment is capable of enriching Foxp3^+^ Tregs in vivo in the spleen and the CNS of EAE mice. Accordingly, p40 treatment also decreased Th1 and Th17 responses in vivo in EAE mice. Next, we investigated mechanisms by which p40 stimulated anti-autoimmune Treg response and attenuated autoimmune Th1 and Th17 responses. Interaction of IL-12 and its receptor IL-12R in the plasma membrane triggers the activation of Janus family of tyrosine kinases, ultimately resulting into the phosphorylation of tyrosine residues of signal transducer and activator of transcription 3 and 4 (STAT3 and STAT4). These tyrosine phosphorylations are responsible for the formation of STAT4/STAT4 homodimer and STAT3/STAT4 heterodimers, which then translocate to the nucleus and bind to IFNγ promoter for the transcription of IFN-γ gene [52]. Similarly, binding of IL-23 to IL-23R leads to the transcription of IL-17 via activation of RORγt [53]. Consistent to the suppression of EAE, p40 inhibited the production of both IFN-γ and IL-17 in MBP-primed T cells, suggesting that the presence of p40 may not favor the interaction of IL-12 with IL-12R and IL-23 with IL-23R to turn on signaling pathways for the production of IFN-γ and IL-17. A successful interaction of IL-12 and IL-23 with their respective receptors leads to the internalization of receptors inside the cell. On the other hand, an unsuccessful interaction leaves the receptor arrested in the membrane, which is then unable to transmit any downstream signaling cascades. Both IL-12R and IL-23R complexes share the same receptor IL-12Rβ1. Interestingly, we found that p40 treatment increased the membrane localization of IL-12Rβ1, but neither IL-12Rβ2 nor IL-23R, in MBP-primed T cells. While IL-12 stimulated the internalization of both IL-12Rβ1 and IL-12Rβ2, p40 suppressed IL-12-mediated internalization of only IL-12Rβ1. Similarly, IL-23 augmented the internalization of both IL-12Rβ1 and IL-23R. However, p40 interfered with the internalization of only IL-12Rβ1 in IL-23-treated T cells. These results demonstrate that p40 is involved in the membrane arrest of IL-12Rβ1 and that p40 is also capable of suppressing IL-12-, IL-23- and p402-mediated IL-12Rβ1 internalization and thereby associated autoimmune signaling pathways. Please see Figure S19 for schematic representation. Accordingly, our studies with Rβ1^−/−^, Rβ2^−/−^ and Rβ1^+/−^/Rβ2^−/−^ mice indicate that IL-12Rβ1 is more critical than IL-12Rβ2 in the induction of EAE and that p40 suppresses EAE via IL-12Rβ1.

However, at present, we do not know mechanisms by which a cell decides to secrete either p40 or p40_2_. Numerous labs [3,54] and we [8,55] have shown the release of IL-12, IL-23, p40, and p40_2_ from antigen-presenting cells such as macrophages, dendritic cells, microglia, etc. However, surprisingly, we have seen that human (LNCaP) and mouse (TRAMP) prostate cancer cells, human (MCF-7) and mouse (4T1) breast cancer cells, and human (Hep3B) and mouse (Hepa) hepatoma cells release excess p40 [16]. Accordingly, we have seen higher level of p40 in serum of prostate cancer patients as compared to age-matched healthy controls. In contrast, levels of IL-12 and p40_2_ are lower in serum of prostate cancer patients as compared to age-matched healthy controls [16]. On the other hand, here, we have observed exactly opposite distribution of p40, IL-12, IL-23, and p402 in serum of MS patients. Therefore, it is likely that IL-12, IL-23 and p402 are produced at greater levels under autoimmune and inflammatory conditions and that p40 is released at higher level during anti-autoimmune and anti-inflammatory conditions. However, further studies are required to find out cellular sources of p40 under inflammatory and anti-inflammatory conditions. For example, recently, we have seen that recombinant p40 is capable of stimulating the production of both pro-inflammatory (TNFα) and anti-inflammatory (IL-10) cytokines from primary lung macrophages [56].

In summary, here, we demonstrate selective decrease of p40 in MS patients and EAE mice and that supplementation of p40 enriches anti-autoimmune Tregs, mitigates autoimmune Th1 and Th17 cells, inhibits the encephalitogenicity of MBP-primed T cells, restores the integrity of BBB and BSB, normalizes the expression of myelin genes in the CNS, and attenuates the clinical symptoms of EAE via suppression of IL-12-, IL-23- and p40_2_-mediated internalization of IL-12Rβ1. These results delineate anti-autoimmune role of p40 that is different from IL-12, IL-23 and p402. Although the disease process of MS is not exactly the same as EAE, our results from MS patients and EAE animals suggest that treatment with p40 may be a new therapeutic strategy against MS.

## Supplementary Material

Supplemental

## Figures and Tables

**Table 1:** Antibodies used in this study

Antibody	Manufacturer	Catalog	Host	Application	Dilution
IL-4	BD Bioscience (PE-tagged)	554435	Rat	FACS	0.5–1 μg/ 106 cells
IFNy	BD Bioscience (PE-tagged)	554412	Mouse	FACS	0.5–1 μg/ 106 cells
IL-17	BD Bioscience (PE-tagged)	559502	Mouse	FACS	0.5–1 μg/ 106 cells
IL-17	Biolegend (APC-647)	506916	Rat	FACS	0.5–1 μg/ 106 cells
IL-12Rβ1	BD Bioscience (PE-tagged)	551974	Mouse	FACS	0.5–1 μg/ 106 cells
IL-12Rβ2	BD Bioscience (PE-tagged)	552819	Hamster	FACS	0.5–1 μg/ 106 cells
Foxp3	eBioscience (PE-tagged)	12–5773-82	Rat	FACS	0.5–1 μg/ 106 cells
Foxp3	Thermofisher (APC-647)	17–5773-82	Rat	FACS	0.5–1 μg/ 106 cells
CD4	BD Bioscience (FITC-tagged)	557307	Mouse	FACS	0.5–1 μg/ 106 cells
CD8	Biolegend (APC/Cy7)	126620	Rat	FACS	0.5–1 μg/ 106 cells
CD3	eBioscience	145–2C11	Hamster	IF	1:100
CD3	eBioscience (FITC-tagged)	11–0033-82	Hamster	FACS	0.5–1 μg/ 106 cells
CD3	Biolegend (BV605)	506916	Rat	FACS	0.5–1 μg/ 106 cells
CD3	eBioscience (PE-tagged)	145–2C11	Hamster	FACS	0.5–1 μg/ 106 cells
GM-CSF	Biolegend (PE/Cy7)	505411	Rat	FACS	0.5–1 μg/ 106 cells

IF, immunofluorescence, FACS, fluorescence-activated cell sorting.

**Table 2:** List of primers used in this study

ICAM-1:	Sense: 5’-CTGGGCTTGGAGACTCAGTG-3’ Antisense: 5’-GTGTCGAGCTTTGGGATGGTA-3’
P-selectin:	Sense: 5’-ACGAGCTGGACGGACCCG-3’ Antisense: 5’-GGCTGGCACTCAAATTTACAG-3’
iNOS:	Sense: 5’-CCCTTCCGAAGTTTCTGGCAGCAGC-3’ Antisense: 5’-GGCTGTCAGAGCCTCGTGGCTTTGG3’
TL-1β:	Sense: 5’-CTCCATGAGCTTTGTACAAGG-3’ Antisense: 5’-TGCTGATGTACCAGTTGGGG-3’
MOG:	Sense: 5’-CCTCTCCCTTCTCCTCCTTC-3’ Antisense: 5’-AGAGTCAGCACACCGGGGTT-3’
MBP:	Sense: 5’-TGGAGAGATTCACCGAGGAGA-3’ Antisense: 5’-TGAAGCTCGTCGGACTCTGAG-3’
PLP:	Sense: 5’-CTTTGCTTCCCTGGTGGCCA-3’ Antisense: 5’-TGTTGGCCTCTGGAACCCCT-3’
CNPase:	Sense: 5’-CTACCCTCCACGAGTGCAAGA-3’ Antisense: 5’-AGTCTAGTCGCCACGCTGTCT-3’
GAPDH:	Sense: 5’-GGTGAAGGTCGGTGTGAACG3’ Antisense: 5’-TTGGCTCCACCCTTCAAGTG-3’
