# Supplementary material for: Corrected Version: IL-12 p40 Monomer is Different from Other IL-12 Family Members to Selectively Inhibit IL-12Rβ1 Internalization and Suppress EAE
Source: J Clin Exp Immunol. Author manuscript; Available in PMC 2026 Sep 16. (PMC13576398; doi:10.33140/jcei.11.03.02)
Supplement: Supplementary file 1 [file NIHMS2198798-supplement-Supplemental.pdf]

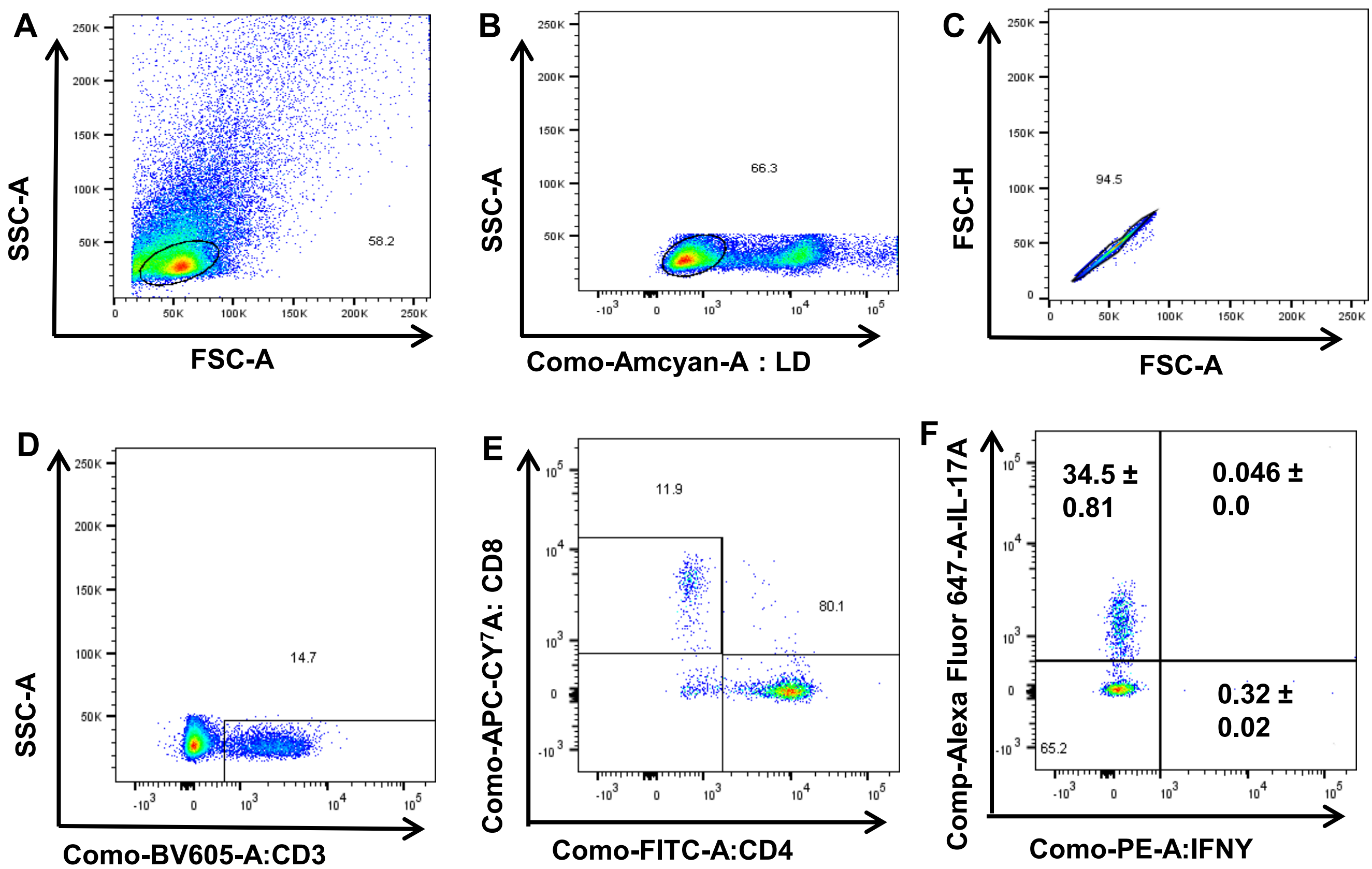

Figure S1. The flow cytometry gating strategy: Lymphocytes were gated based on side scatter (SSC-A) versus forward scatter (FSC-A) (A), dead cells excluded with Zombie Aqua™ Fixable Viability Kit (B), and singlets were selected from the FSC-H versus FSC-A dot plot (C), then divided into T cells on the basis of surface expression of CD3 (D). The dot plot on the lower right and upper left show a clear demarcation of CD4 and CD8 cell populations (E). IL-17^{+} and IFNγ^{+} cells were analyzed from CD4 population of cells (F).

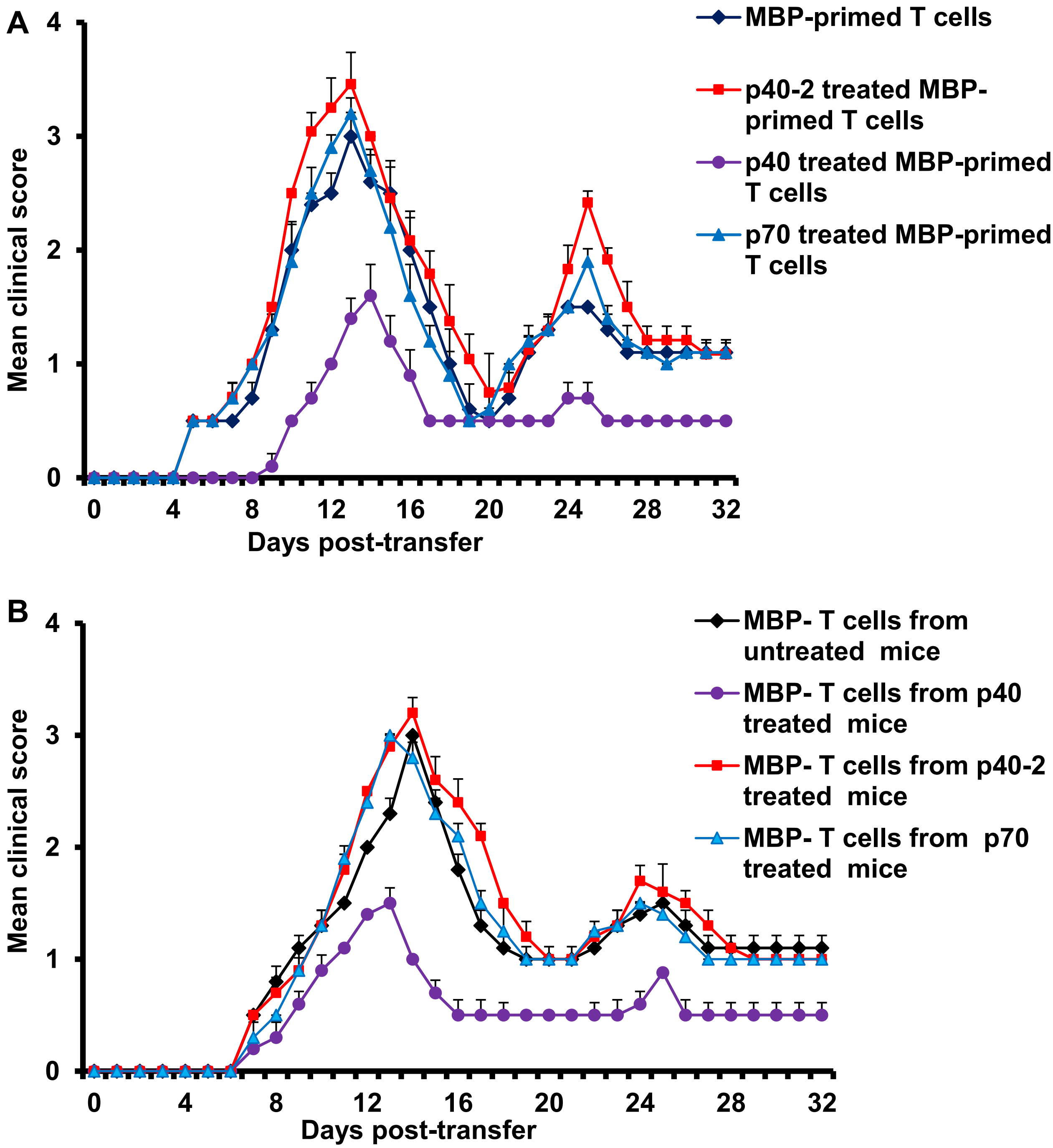

Figure S2. Effect of p40 on the encephalitogenicity of MBP-primed T cells. A, LNC isolated from MBP-immunized donor mice were treated with MBP in the presence or absence of 10 ng/ml p40, $p40_2$ or IL-12. After 4 d, these LNCs were transferred to naïve female SJL/J mice by adoptive transfer. B, Donor mice were immunized with MBP, IFA, and *M. tuberculosis.* On 2nd day of immunization, mice were treated with 200 ng/mouse p40, $p40_2$ or IL-12 via i.p. injection. On day 12 of immunization, mice were sacrificed, and total LNC were further primed with MBP for 4 days followed by adoptive transfer of these LNCs to naïve recipient mice. Six mice were used in each group.

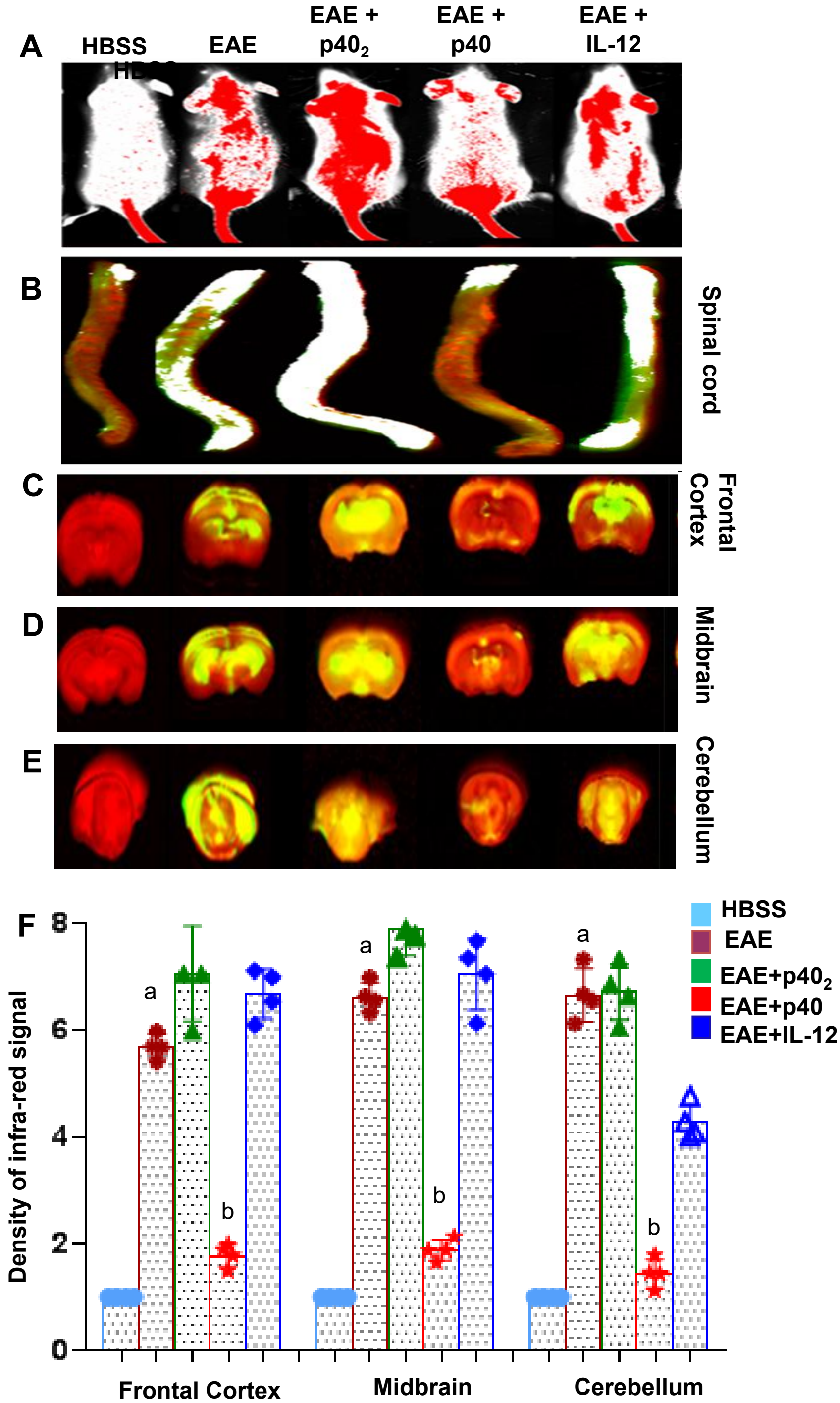

Figure S3. Effect of p40 on the integrity of BBB and BSB and the CNS infiltration of mononuclear cells in EAE mice. A) Control and different groups of EAE mice (n=4 in each group) received 200 ml of 20 mM Alexa 680-SE-NIR dye (Life Technologies) via tail vain on 14 dpt (acute phase). After 2 h, mice were scanned in Odyssey (ODY-0854, Licor-Inc) infrared scanner at 700- and 800-nm channels. Mice were perfused with 4% paraformaldehyde. Spinal cord (B), frontal cortex (C), midbrain (D) and cerebellum (E) were scanned in Odyssey infrared scanner. The red background came from 800nm filter, whereas the green signal was from Alexa 680 dye at 700 nm channel. F) The density of the Alexa 680 signal in different parts of the brain was quantified with the help of Quantity One, version 4.6.2 software using the volume contour tool analysis module. Data are expressed as the mean ± SEM of four mice per group. ^{a}*p < 0.001* vs control; ^{b}*p < 0.001* vs EAE.

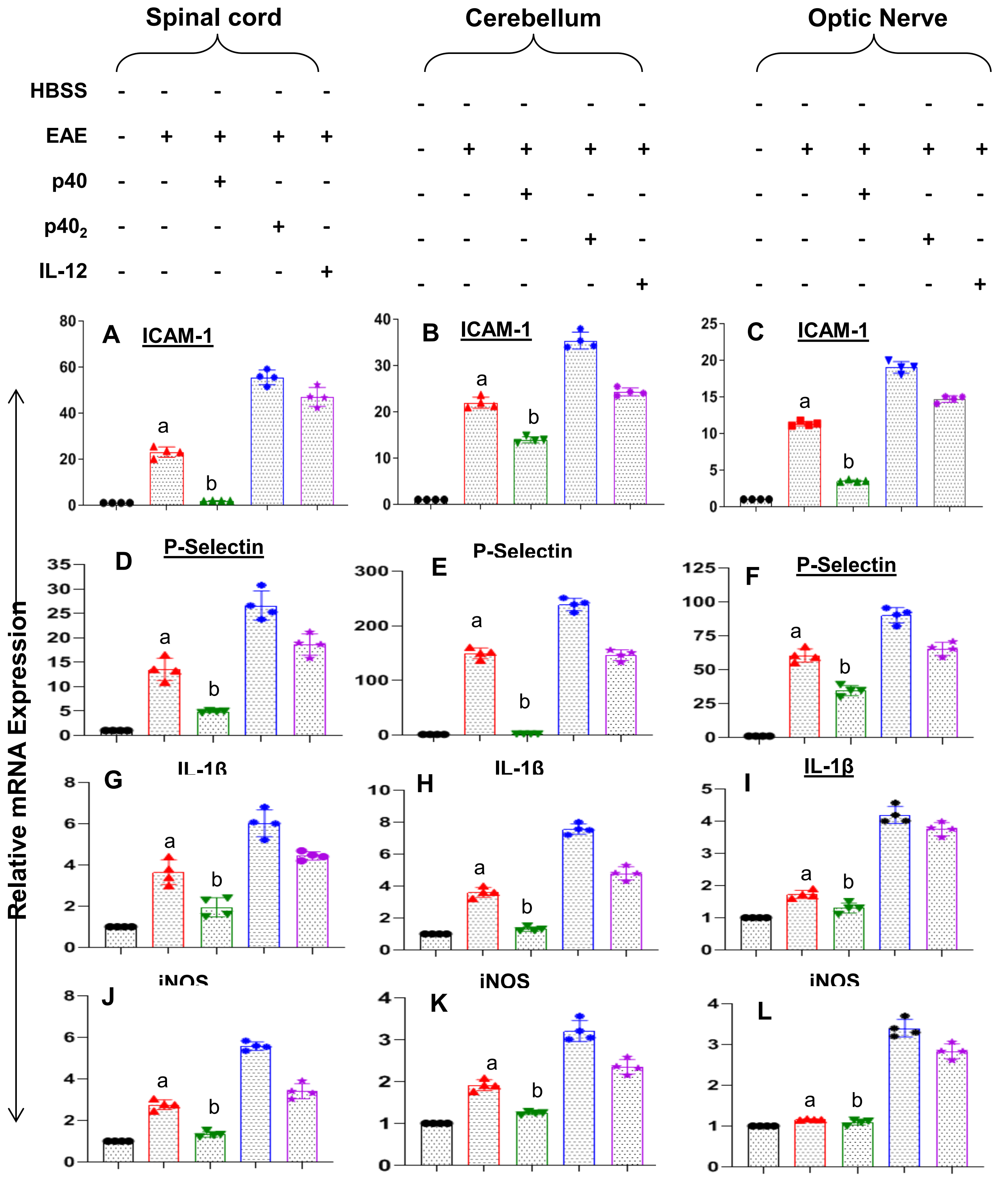

Figure S4. Effect of p40 on the expression of adhesion molecules and proinflammatory markers in spinal cord, cerebellum and optic nerve of EAE mice. Spinal cord (A, D, G, & J), cerebellum (B, E, H, & K) and optic nerve (C, F, I, & L) of normal, EAE (14 dpt) and p40-, $p40_2$- or IL-12-treated EAE (14 dpt receiving these cytokines on 8 dpt) mice were analyzed for the mRNA expression of ICAM-1 (A-C), P-selectin (D-F), IL-1β (G-I), and iNOS (J-L) by real-time PCR. On 8 dpt, mice were treated with 200 ng/mouse p40, $p40_2$ or IL-12 via i.p. injection. Results are mean ± SEM of four mice per group. ^{a}p < *0.001* vs control; ^{b}p < *0.001* vs EAE.

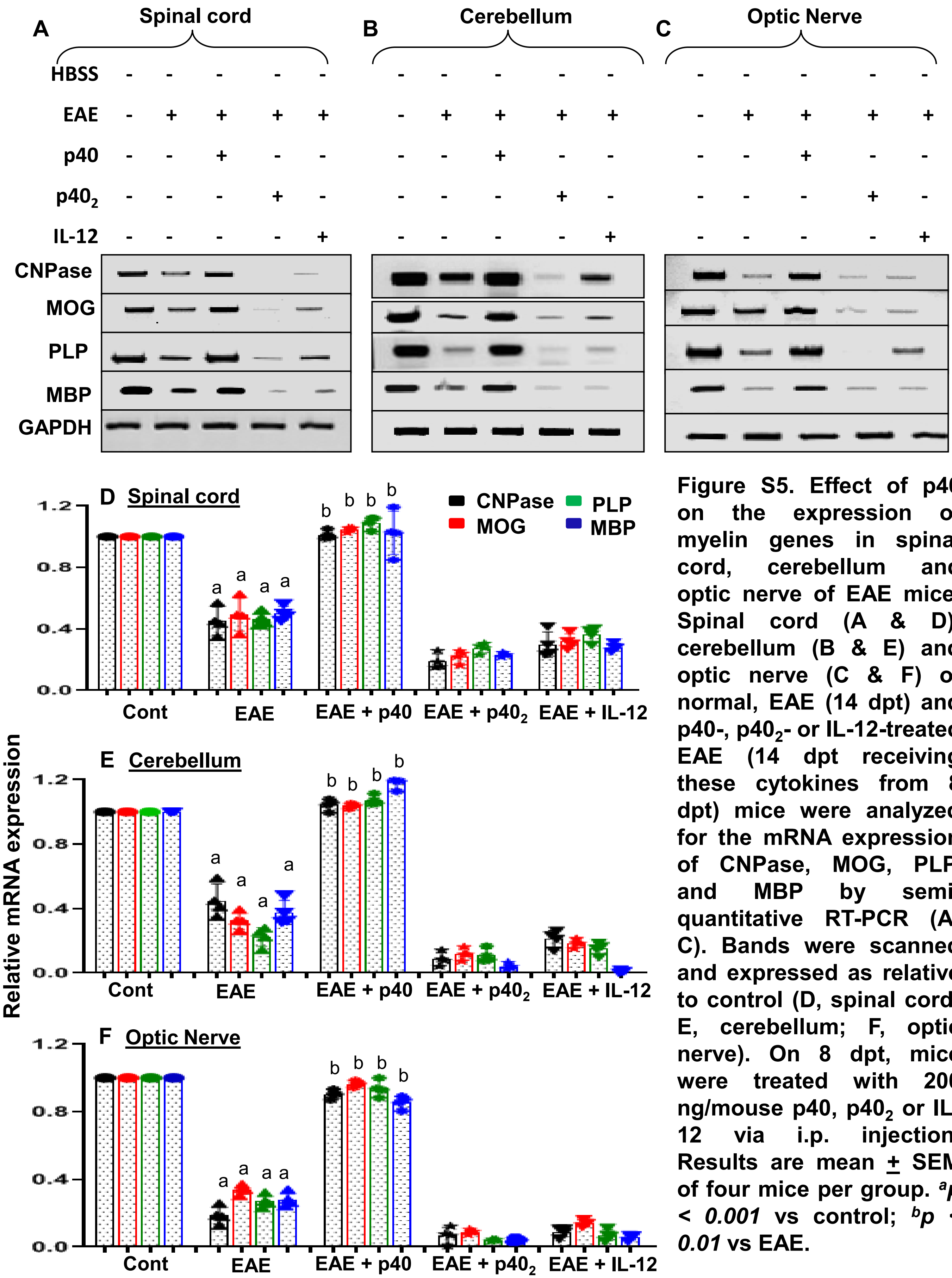

Figure S5. Effect of p40 on the expression of myelin genes in spinal cord, cerebellum and optic nerve of EAE mice. Spinal cord (A & D), cerebellum (B & E) and optic nerve (C & F) of normal, EAE (14 dpt) and p40-, $p40_2$- or IL-12-treated EAE (14 dpt receiving these cytokines from 8 dpt) mice were analyzed for the mRNA expression of CNPase, MOG, PLP, and MBP by semi-quantitative RT-PCR (A-C). Bands were scanned and expressed as relative to control (D, spinal cord; E, cerebellum; F, optic nerve). On 8 dpt, mice were treated with 200 ng/mouse p40, $p40_2$ or IL-12 via i.p. injection. Results are mean ± SEM of four mice per group. $^{a}p < 0.001$ vs control; $^{b}p < 0.01$ vs EAE.

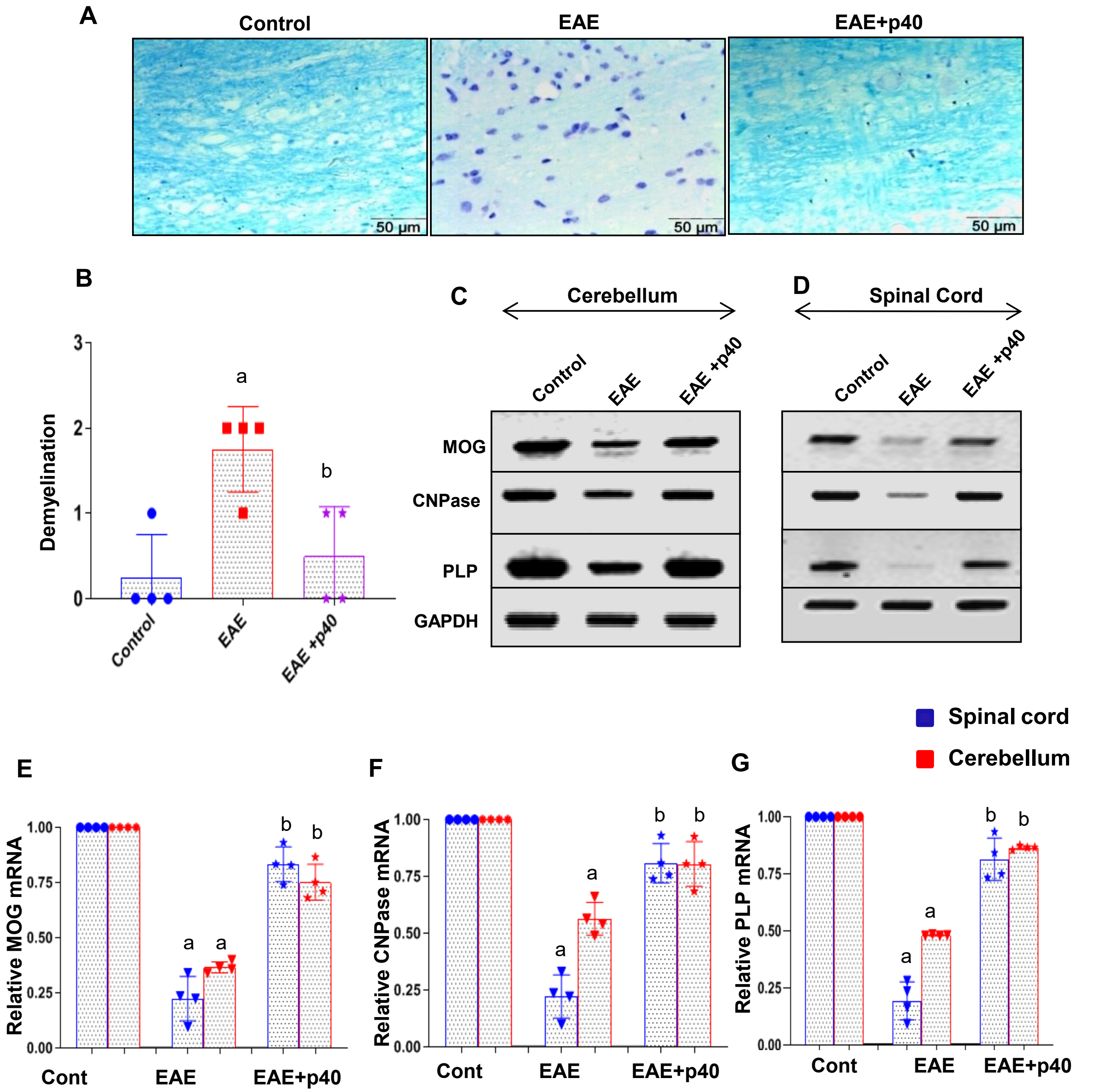

Figure S6. Effect of p40 on demyelination in the CNS of mice with Ch-EAE. MOG-induced chronic EAE mice received weekly p40 treatment (200 ng/mouse) from 10 dpi via i.p. injection. On 18 dpi, cerebellar sections were stained with Luxol fast blue. Digital images were collected under bright field setting using an x 40 objective (A). Demyelination in cerebellum (B) was represented quantitatively by using a scale as described in materials and methods. Data are expressed as the mean ± SEM of four mice per group. $^{a}p < 0.001$ vs control; $^{b}p < 0.001$ vs EAE. The mRNA expression of MOG, CNPase and PLP was examined in cerebellum (C) and spinal cord (D) by RT-PCR and real-time PCR (E, MOG; F, CNPase; G, PLP). Data are expressed as the mean ± SEM of four mice per group. $^{a}p < 0.001$ vs control; $^{b}p < 0.001$ vs EAE.

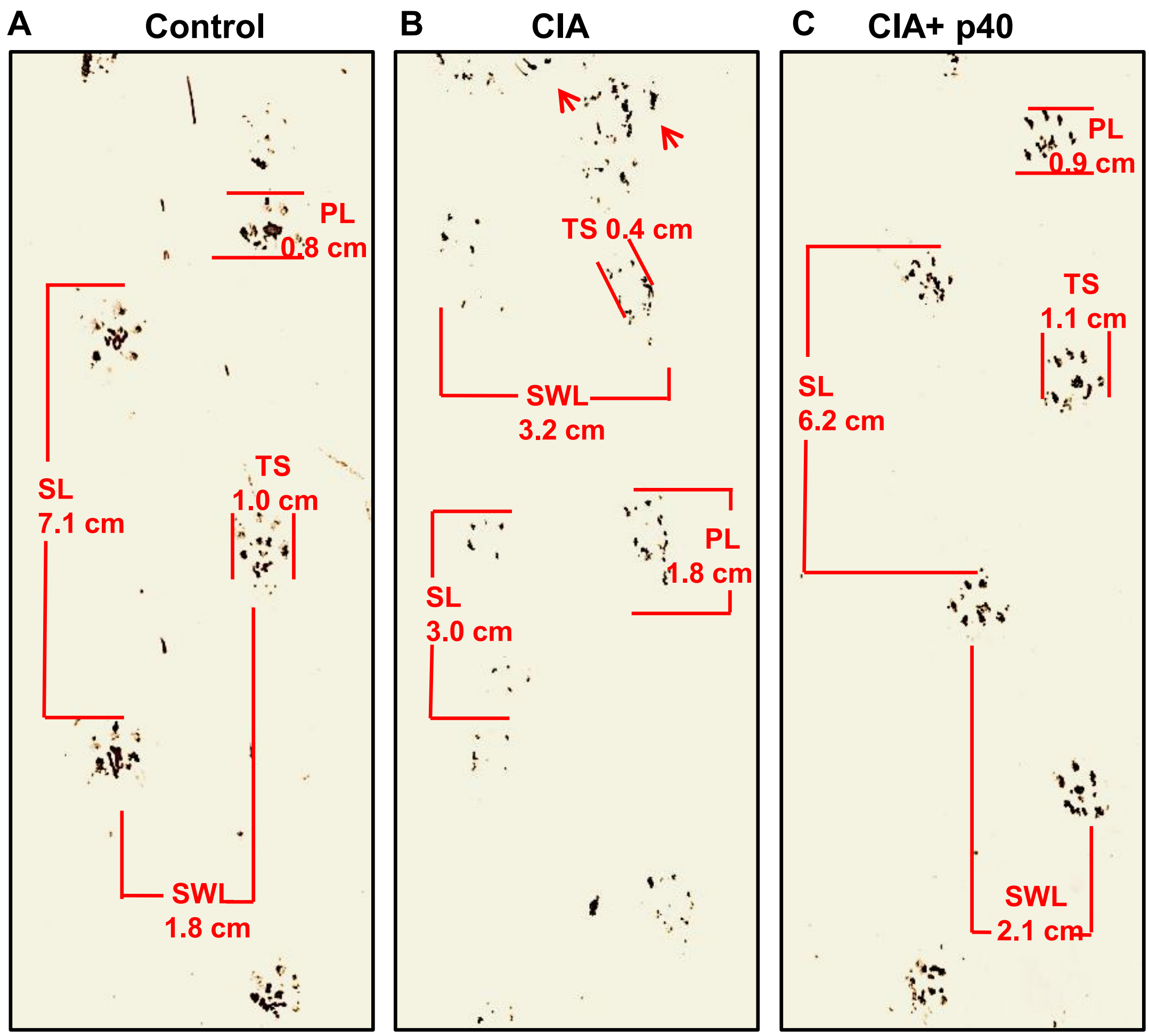

Figure S7. Footprint analysis of mice with CIA after treatment with p40. On the walking track, we applied white paper strips and obtained the footprints of mice of different groups (A, control; B, CIA; C, CIA+p40) on paper using black ink. A total of 30-40 steps for each group were determined. Four different footprint measurements (SL, PL, SWL, and TS) were calculated in centimeters from the recorded prints of mice. Six mice (n=6 per group) were used in two independent experiments.

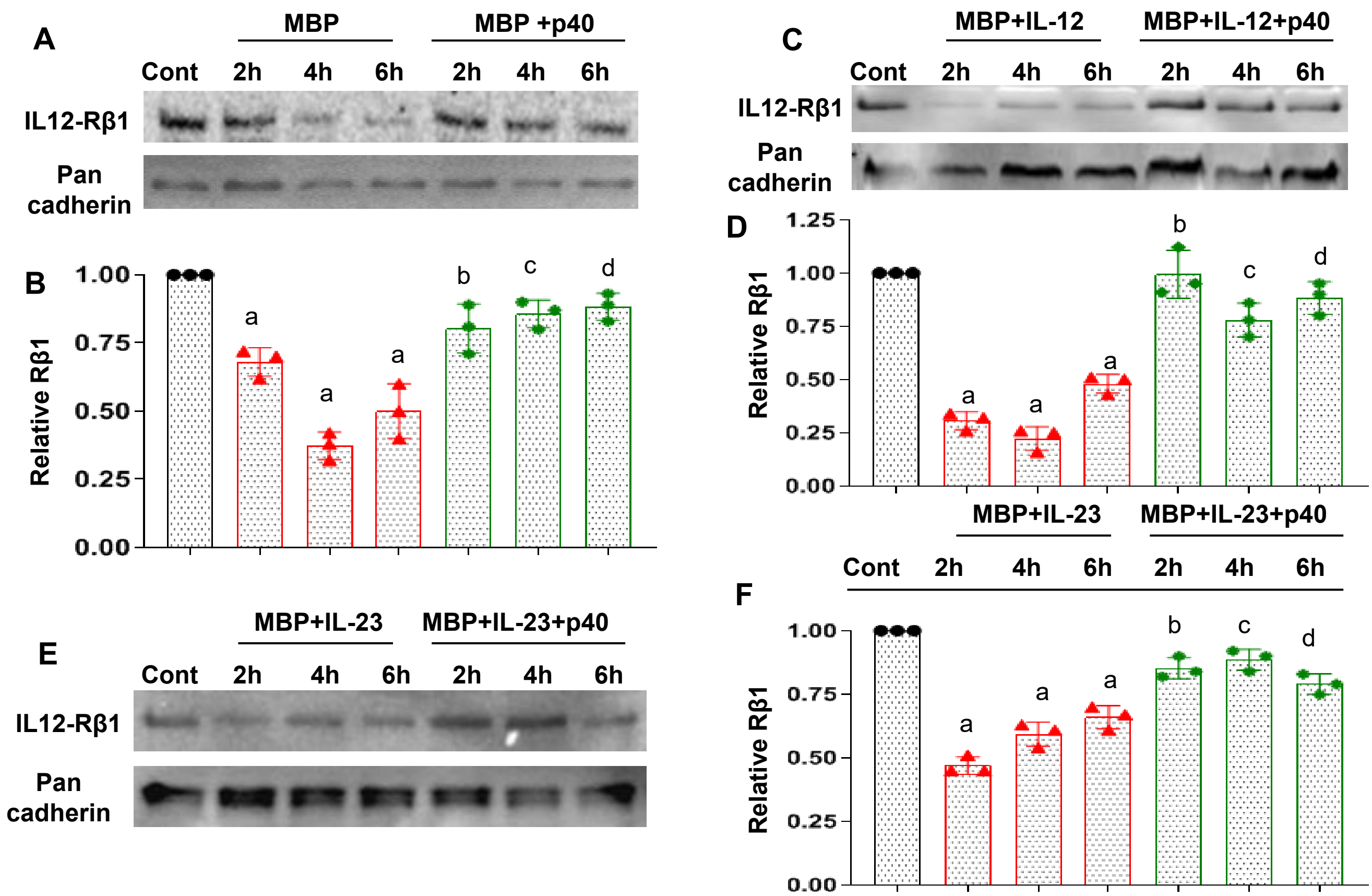

Figure S8. The p40 treatment inhibits the internalization of IL-12Rβ1 in MBP-primed T cells. After different h of stimulation with MBP and MBP+p40 (A&B), MBP+IL-12 and MBP+IL-12+p40 (C&D), MBP+IL-23 and MBP+IL-23+p40 (E&F), the level of IL-12Rβ1 was monitored in cell membrane of non-adherent splenocytes by Western blot. Cadherin was run as a marker for cell membrane. Bands were scanned and values (IL-12Rβ1/cadherin) are presented as relative to control. Data are expressed as the mean ± SD of three independent experiments. $^{a}p < 0.01$ vs control; $^{b}p < 0.01$ vs 2h; $^{c}p < 0.01$ vs 4h; $^{d}p < 0.05$ vs 6h. IL-12, 10 ng/ml; IL-23, 10 ng/ml; p40, 10 ng/ml.

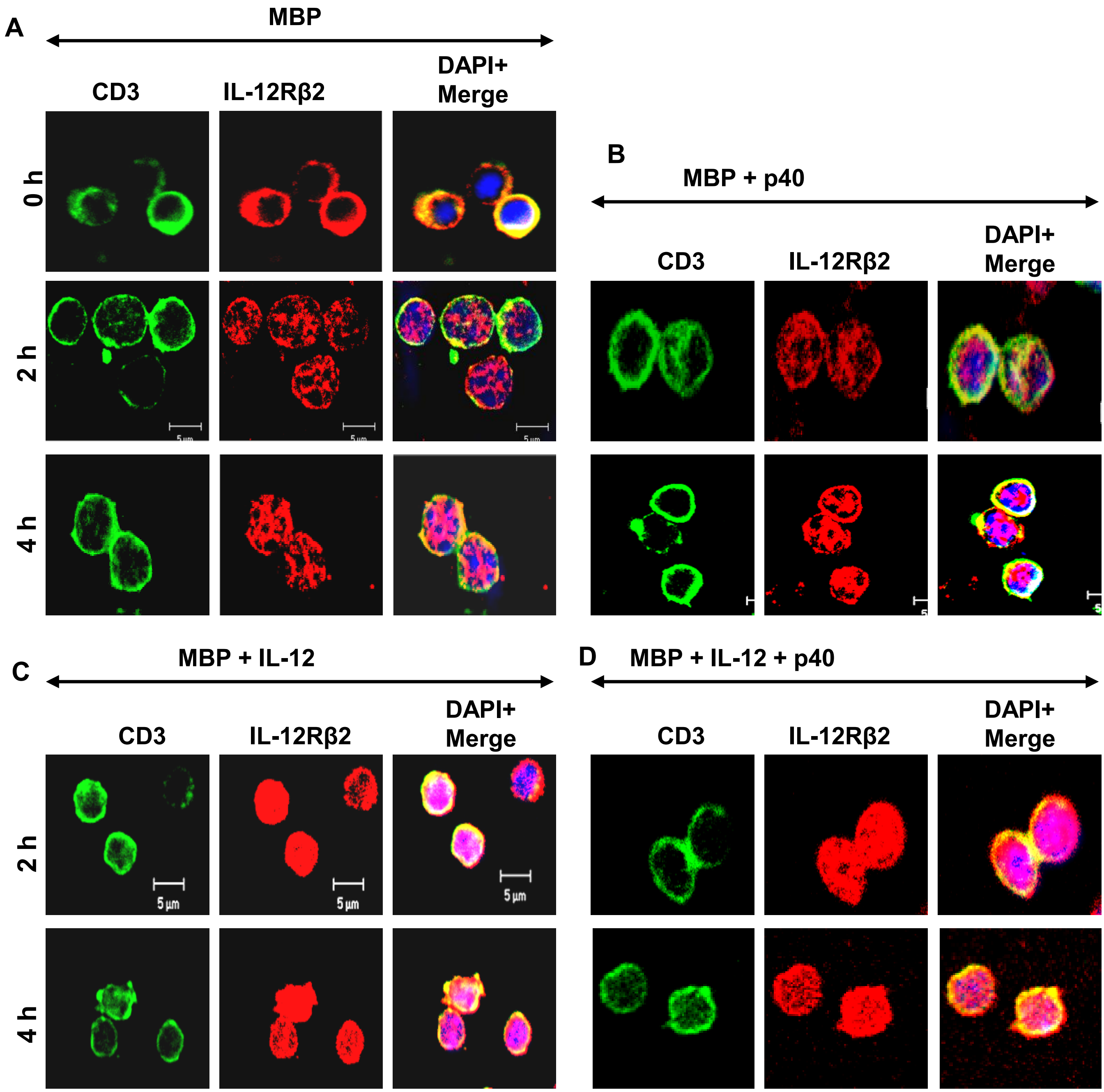

Figure S9. The p40 does not modulate the internalization of IL-12Rβ2 in MBP-primed T cells. Splenocytes isolated from MBP-immunized donor mice were re-stimulated with MBP (A) in the presence or absence of p40 (B), IL-12 (C) and p40+IL-12 (D) for different time periods followed by double-label immunofluorescence of CD3 and IL-12Rβ2. Cells were treated with p40 thirty min before IL-12. At 0 min of MBP re-stimulation (A), IL-12Rβ2 was present mainly on the membrane. However, at 2 h or 4 h, IL-12Rβ2 was internalized. This internalization was not inhibited by p40 (B). Greater degree of IL-12Rβ2 internalization was observed after IL-12 treatment (C). Again, p40 pretreatment was unable to block the internalization of IL-12Rβ2 in IL-12-treated cells. IL-12, 10 ng/ml; p40, 10 ng/ml. Results represent three independent experiments.

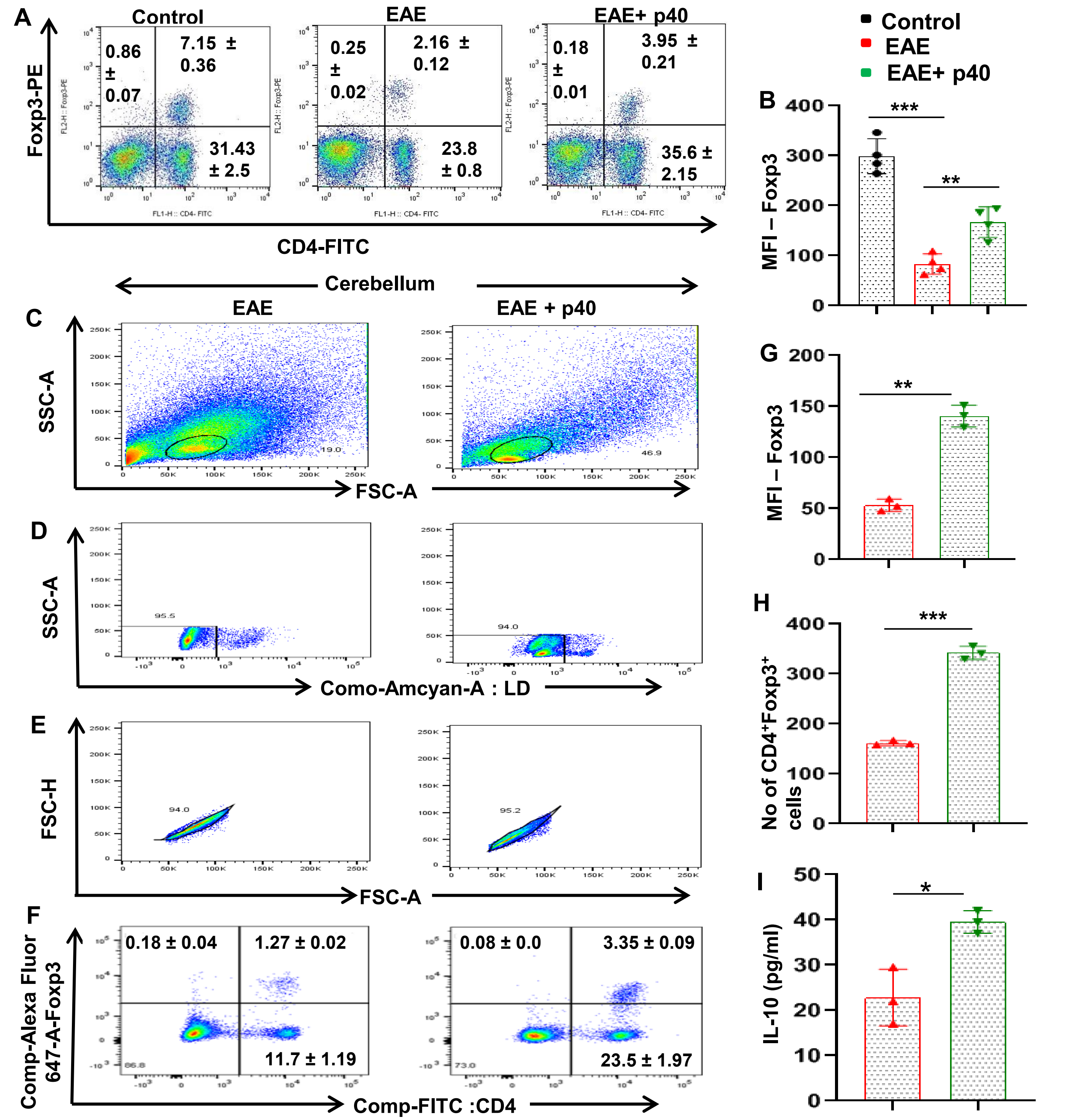

Figure S10. Enrichment of Tregs *in vivo* in the spleen and cerebellum of EAE mice. Adoptively-transferred EAE mice received weekly treatment of p40 (200 ng/mouse) via i.p. injection from 8 dpt. On 16 dpt, splenocytes were analyzed by FACS for CD4 and Foxp3 (A) followed by determining MFI of Foxp3 in $CD4^+$ cells (B). Results are mean ± SEM of four mice (n=4) per group. $^{**}p<0.01$; $^{***}p<0.001$. Single-cell suspensions from cerebellum were stained with Zombie Aqua™ Fixable Viability Kit (Biolegend). Cells were washed with FACS buffer (Thermo-Scientific) and stained with CD4-FITC and CD8-APC-Cy7 (Biolegend) for extracellular stains. The eBioscience™ Foxp3/Transcription Factor Staining Buffer set (Thermo-scientific) was used for intracellular staining of Foxp3-APC. Multicolor flow cytometric analyses were performed using LSRFortessa analyzer (BD Biosciences) and analyzed using FlowJo Software (v10) (C, SSC-A versus FSC-A; D, dead cell exclusion; E, selection of singlets from the FSC-H versus FSC-A dot plot; F, analysis of $Foxp3^+$ cells from $CD4^+$ cells; G, MFI of Foxp3 in $CD4^+$ population; H, number of $CD4^+Foxp3^+$ T cells out of ~1680 $CD4^+$ T cells). Cerebellum of two mice were pooled together giving rise to three different sets from a total of six mice per group. Therefore, data are mean ± SD of three different sets. $^{***}p<0.001$. I) Level of IL-10 was measured in the cerebellum by ELISA. Data are mean ± SEM of three mice per group. $^{*}p<0.05$.

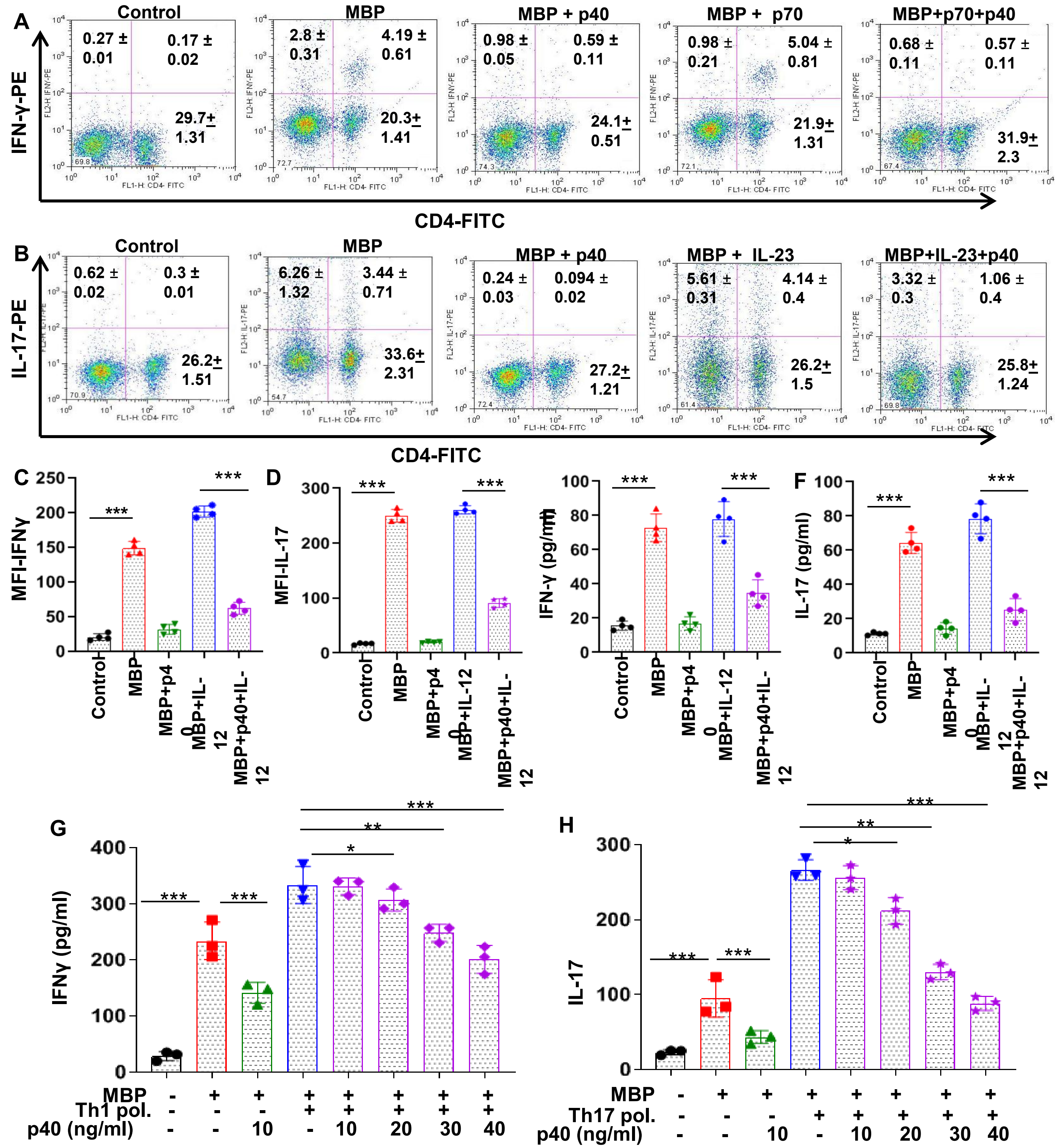

Figure S11. Suppression of Th1 and Th17 responses by p40. Splenocytes isolated from MBP-immunized donor mice were re-stimulated with MBP in the presence or absence of p40, IL-12 and p40+IL-12 for 48 h followed by FACS analysis for CD4 and IFNγ (A). Splenocytes were re-stimulated with MBP in the presence or absence of p40, IL-23 and p40+IL-23 for 48 h followed by FACS analysis for CD4 and IL-17 (B). The MFI of IFNγ (C) and IL-17 (D) in $CD4^+$ population was calculated by using the CellQuest software. The level of IFNγ (E) and IL-17 (F) was monitored in supernatants by ELISA. Data are mean ± SD of four different experiments. Cells were treated with p40 30 min before either IL-12 or IL-23. IL-12, 10 ng/ml; IL-23, 10 ng/ml; p40, 10 ng/ml. Splenocytes were treated with p40 followed by re-stimulation with MBP in the presence of either Th1 (G) or Th17 (H) polarization. After 48 h, levels of IFNγ (G) and IL-17 (H) were monitored by ELISA. Results are mean ± SD of three different experiments. $^{*}p < 0.05$, $^{**}p < 0.01$ & $^{***}p < 0.001$ by two-sample t-tests.

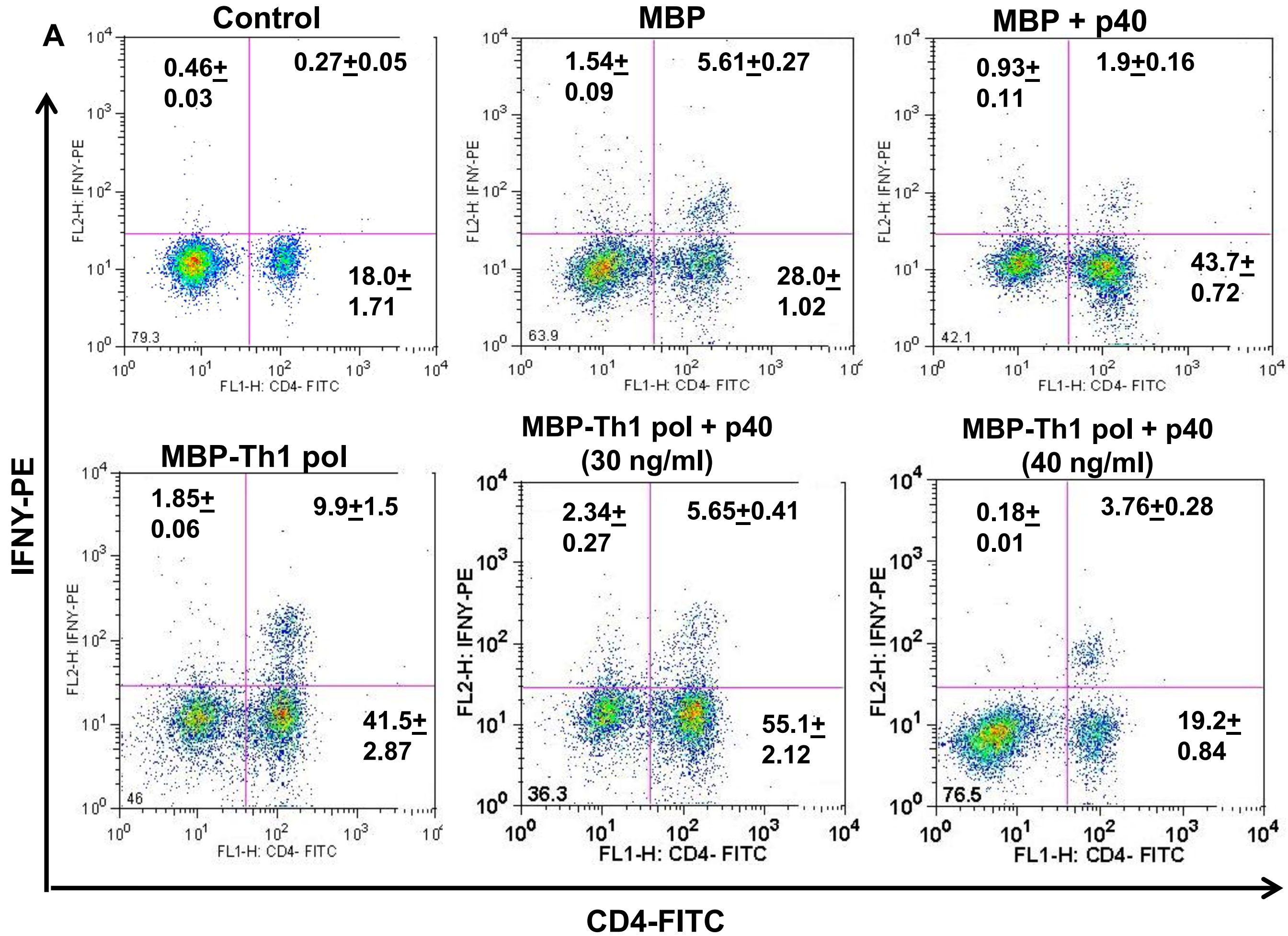

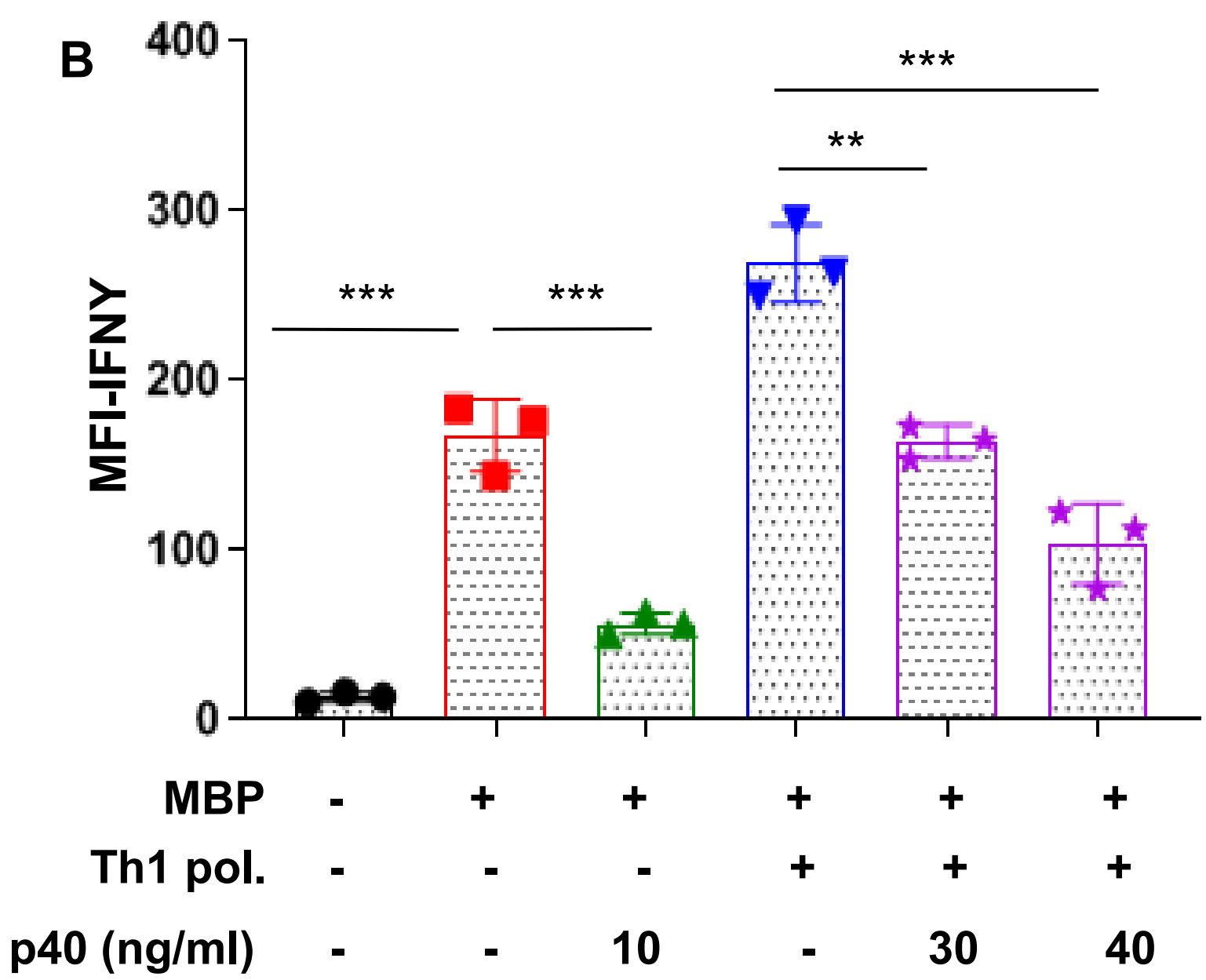

Figure S12. Effect of p40 on the expression of IFNγ under Th1 polarizing condition. Splenocytes isolated from MBP-immunized donor mice were re-stimulated with MBP in the presence or absence of p40. For Th1 conditioning, splenocytes also received 4 ng/ml IL-12 (R&D) plus 50 U IL-2 (R&D). After 72 h, non-adherent were analyzed by FACS in LSRFortessa analyzer (BD Biosciences) for CD4 and IFNγ (A). The MFI of IFNγ (B) in $CD4^+$ population was calculated by using the CellQuest software. Data are mean ± SD of three different experiments. $^{***}p < 0.001$; $^{**}p < 0.01$.

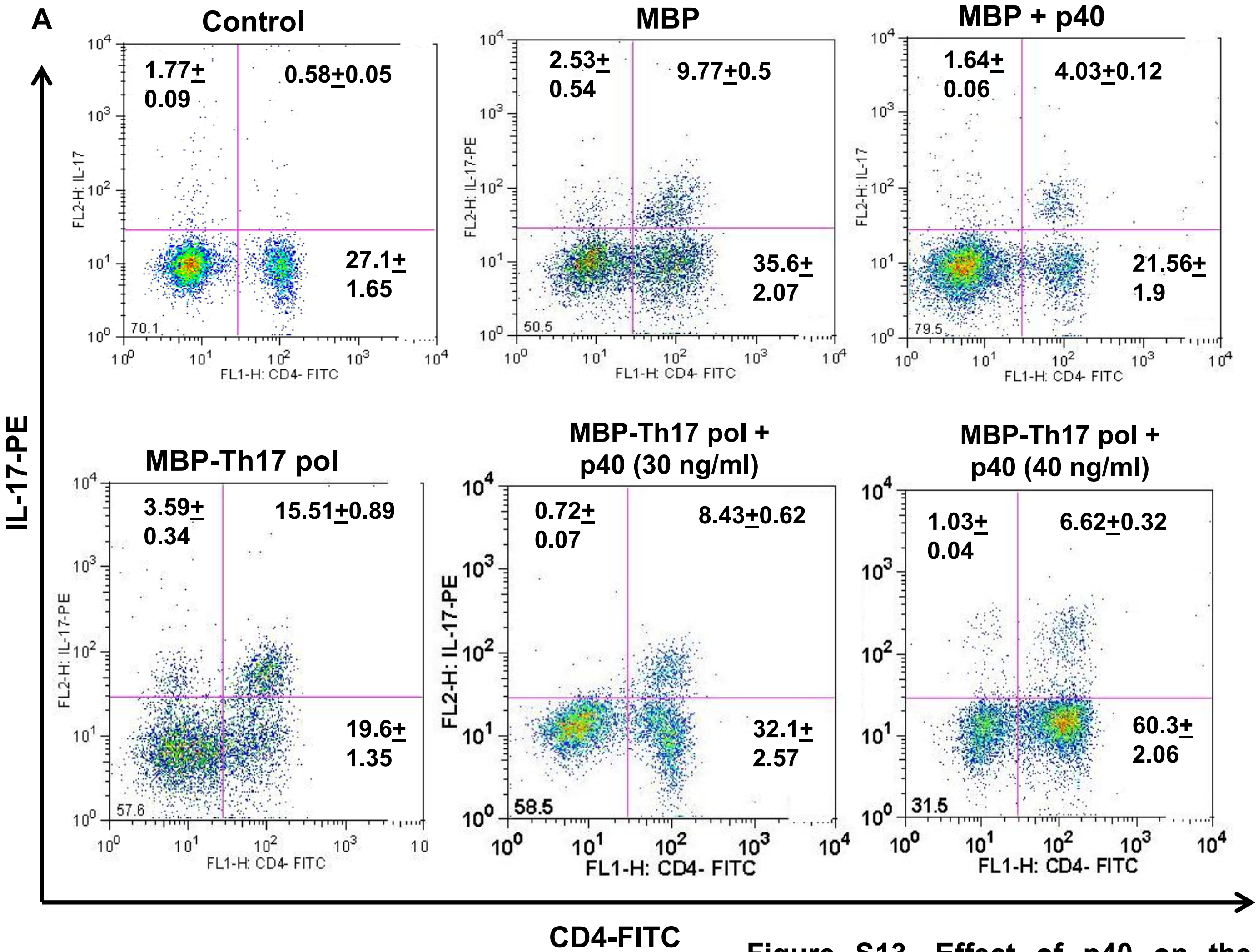

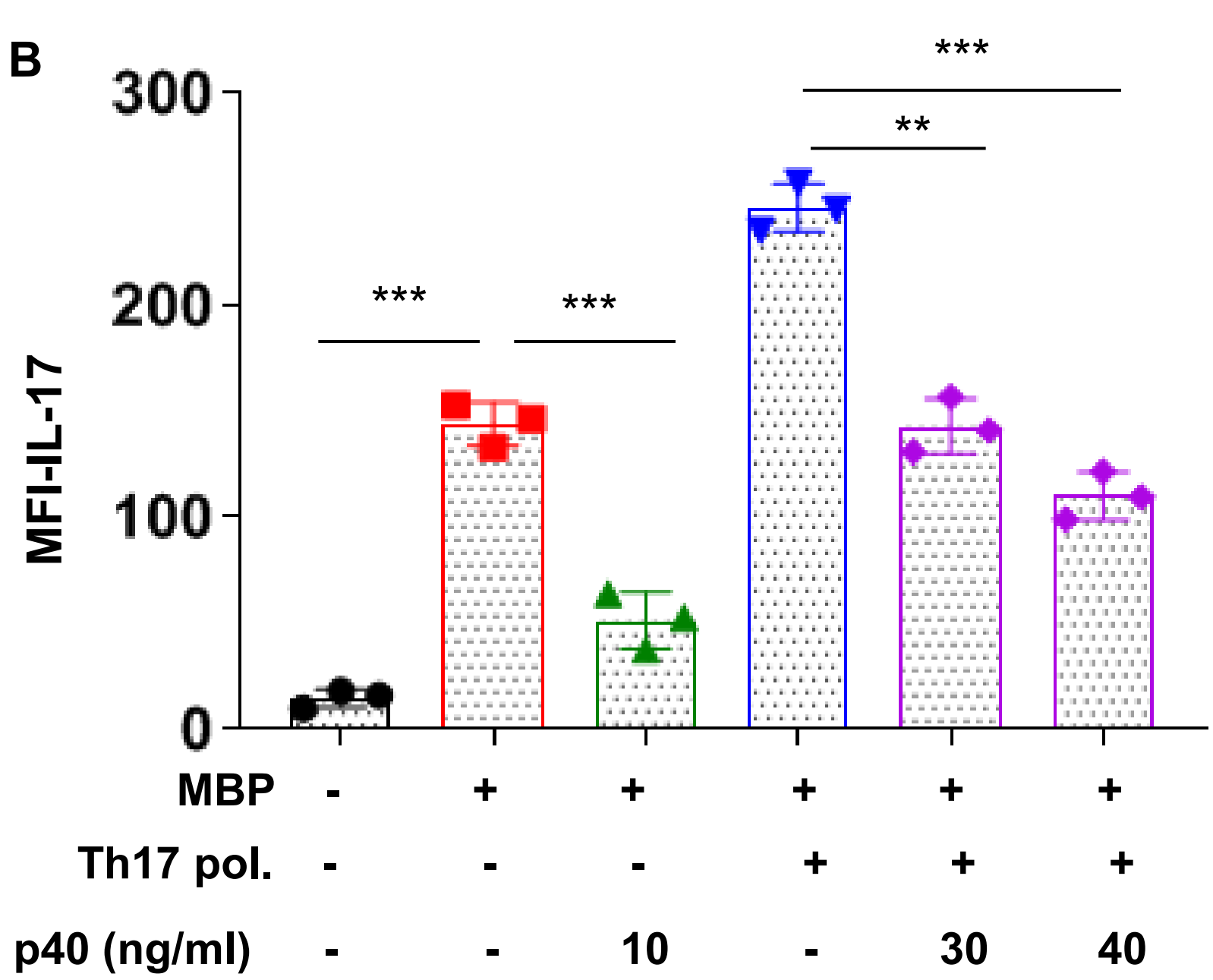

Figure S13. Effect of p40 on the expression of IL-17 under Th17 polarizing condition. Splenocytes isolated from MBP-immunized donor mice were re-stimulated with MBP in the presence or absence of p40. For Th17 conditioning, splenocytes also received 5 μg/ml anti-IFN-γ antibody (eBioscience), 30 ng/ml IL-6 (R&D), 2 ng/ml TGF-β (R&D), and 50 U IL-2. After 72 h, non-adherent cells were analyzed by FACS in LSRFortessa analyzer (BD Biosciences) for CD4 and IL-17 (A). The MFI of IL-17 (B) in $CD4^+$ population was calculated by using the CellQuest software. Data are mean ± SD of three different experiments. $^{***}p < 0.001$; $^{**}p < 0.01$.

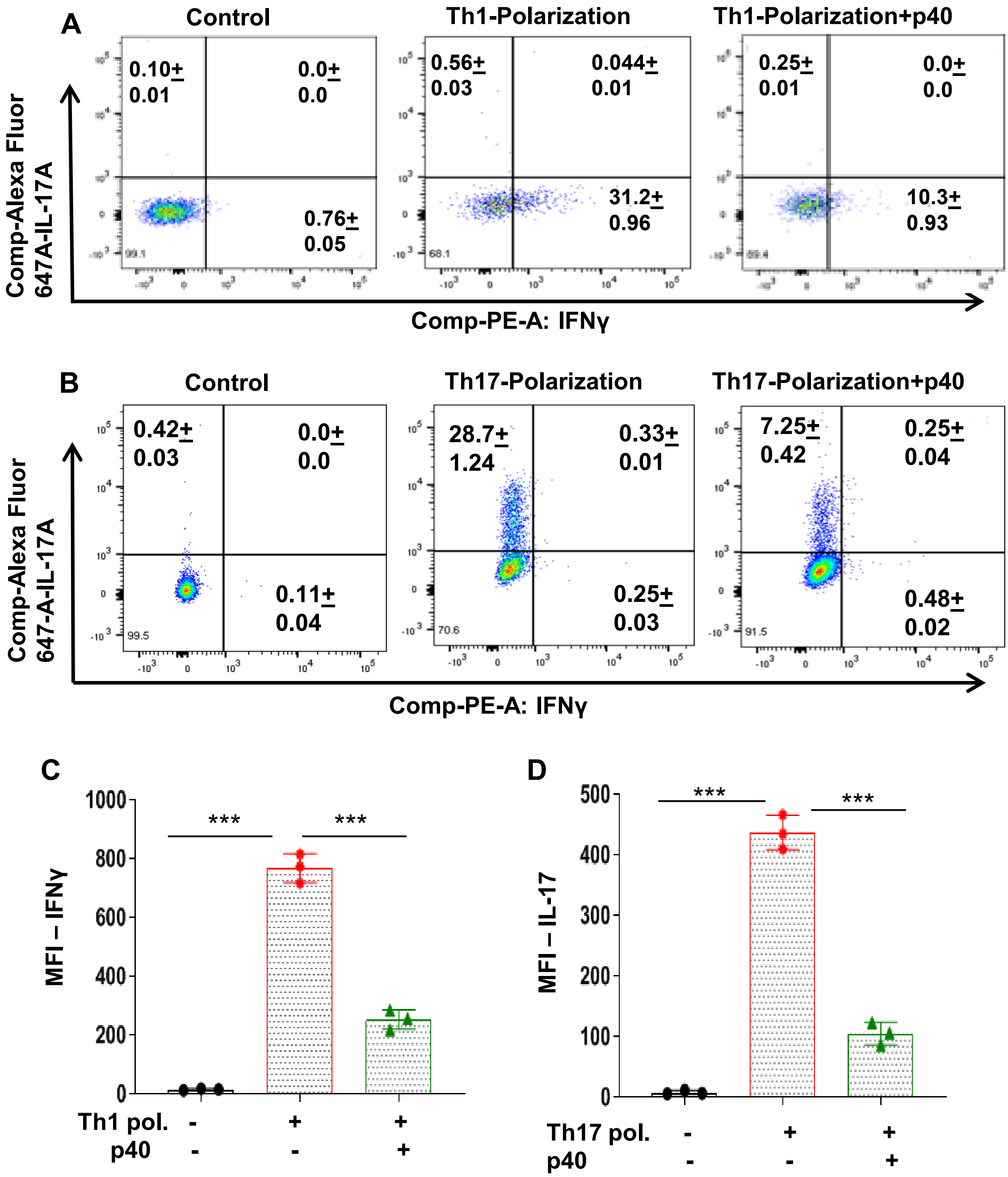

Figure S14. Effect of p40 on T helper cell differentiation towards Th1 or Th17 cells during priming *in vitro*. CD4⁺ T cells were cultured in plates coated with 1 µg/ml anti-CD3 antibodies (BD Bioscience, San Jose, CA) and treated with anti-CD28 antibodies (BD Bioscience) at a concentration of 1 µg/ml for 2 d. For polarization to either Th1 (A) or Th17 (B), particular set of cytokines or antibodies were added in the presence or absence of p40 (30 ng/ml) for 3 d followed by 2-color FACS in LSRFortessa analyzer (BD Biosciences) for detecting IFNγ and IL-17 at the same time. Naïve CD4⁺ T cells were induced to differentiate into Th1 type by adding 4 ng/ml IL-12 (eBioscience) plus 50 U recombinant human IL-2 (rhIL2, eBioscience) and into Th17 cells by addition of 5 µg/ml anti–IFN-γ (R&D Systems), 30 ng/ml rhIL-6, 2 ng/ml rhTGF-β (eBioscience), and 50 U rhIL-2. The MFI of IFNγ (C) and IL-17 (D) in CD4⁺ population was calculated by using the CellQuest software. Data are mean ± SD of three different experiments. $^{***}p < 0.001$.

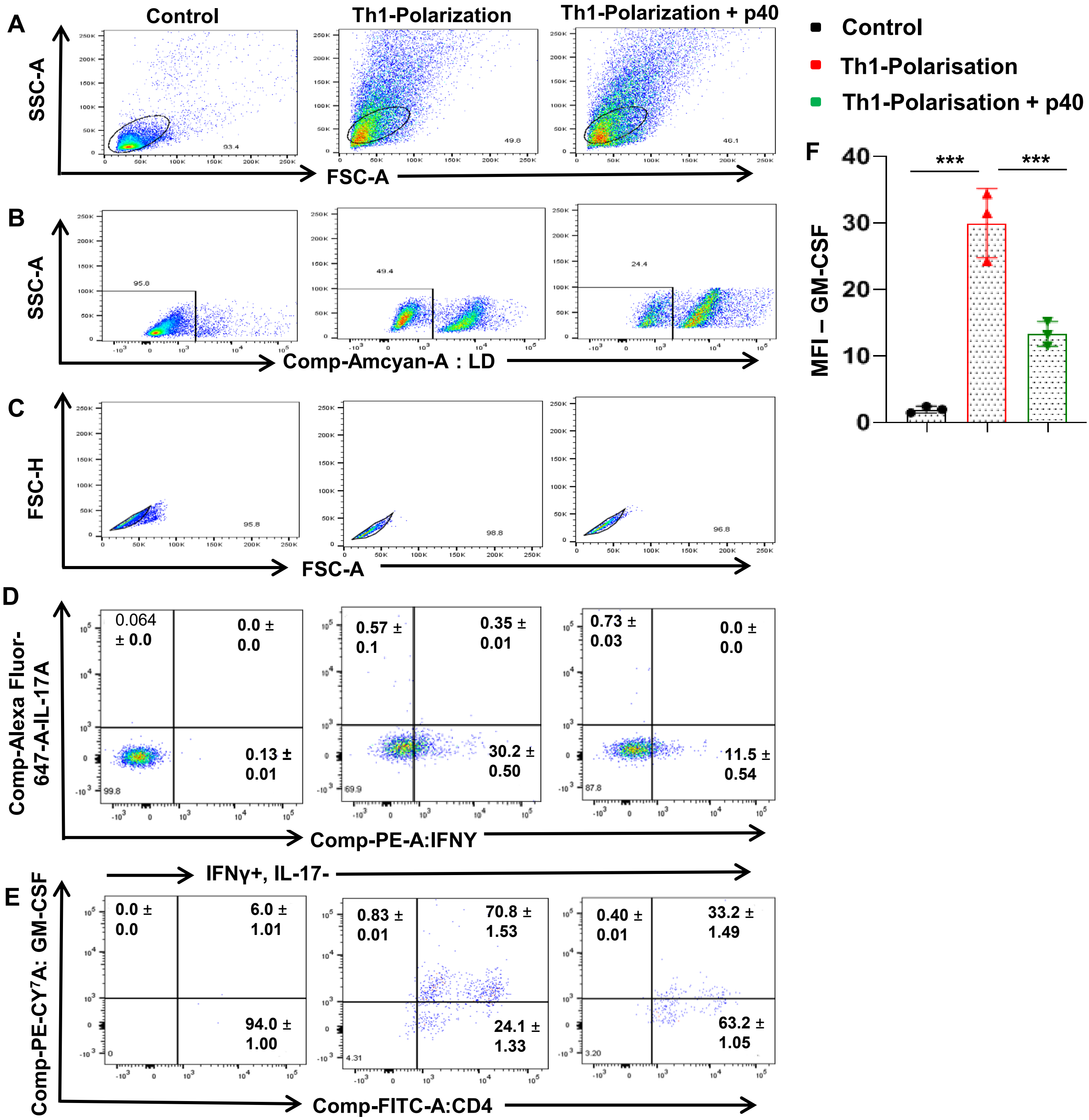

Figure S15. Effect of p40 on GM-CSF in Th1 cells. CD4$^+$ T cells were cultured in plates coated with 1 µg/ml anti-CD3 antibodies (BD Bioscience, San Jose, CA) and treated with anti-CD28 antibodies (BD Bioscience) at a concentration of 1 µg/ml for 2 d. For polarization to Th1, 4 ng/ml IL-12 (eBioscience) plus 50 U recombinant human IL-2 (rhIL2, eBioscience) were added in the presence or absence of p40 (30 ng/ml) for 3 d followed by multicolor FACS in LSRFortessa analyzer (BD Biosciences) for detecting GM-CSF in IFNγ$^+$IL-17$^-$ CD4$^+$ T cells. Results were analyzed using FlowJo Software (v10) (A, SSC-A versus FSC-A; B, dead cell exclusion; C, selection of singlets from the FSC-H versus FSC-A dot plot; D, analysis of IFNγ$^+$IL-17$^+$ and/or IFNγ$^+$IL-17$^-$ cells from CD4$^+$ cells; E, analysis of GM-CSF$^+$ cells from IFNγ$^+$IL-17$^-$ cells). The MFI of GM-CSF in IFNγ$^+$IL-17$^-$ cells (F) was calculated by using the CellQuest software. Data are mean ± SD of three different experiments. ****p < 0.001*.

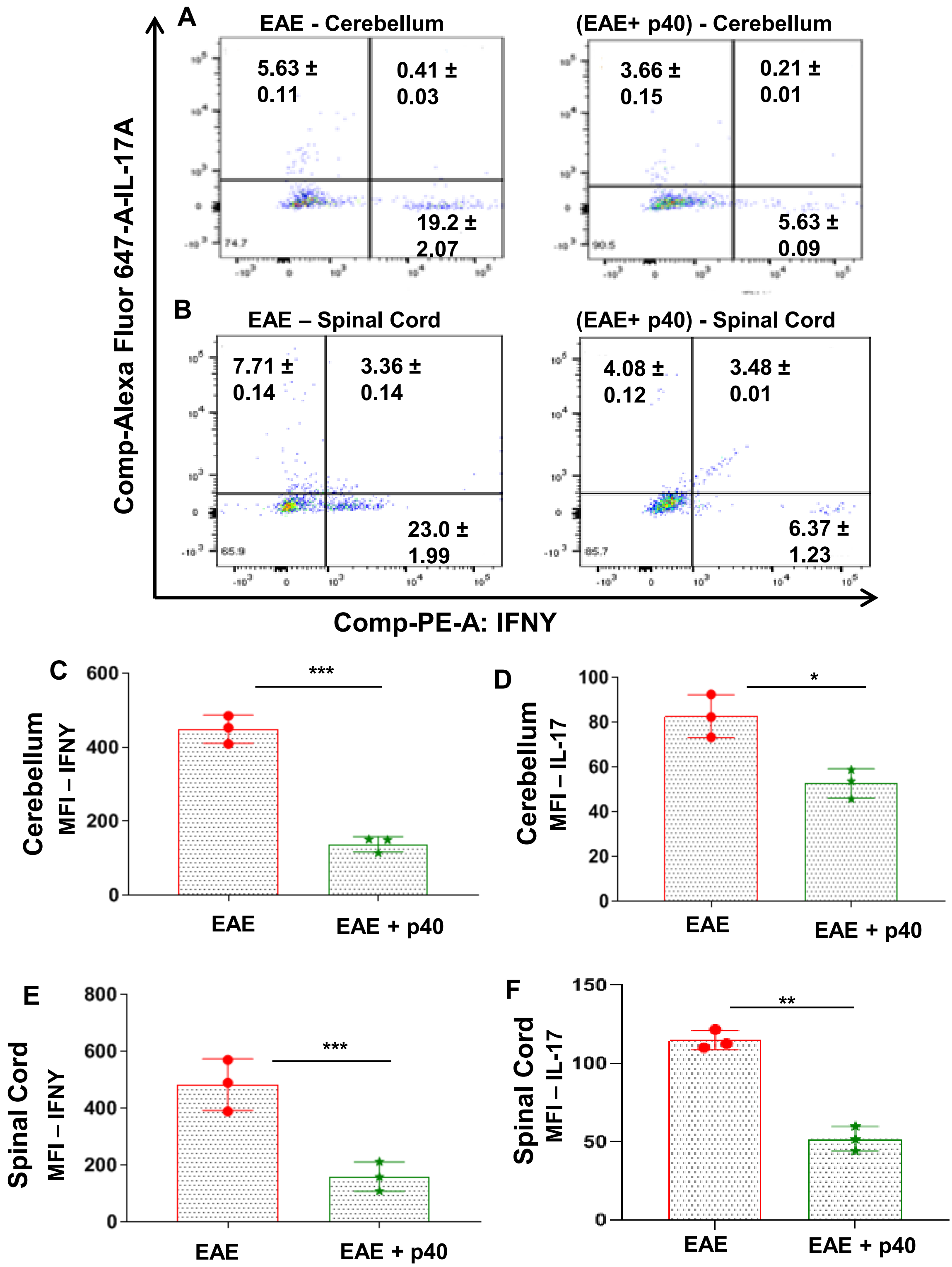

Figure S16. Effect of p40 on the differentiation of CD4$^+$ T cells towards Th1 and Th17 cells in CNS infiltration of mononuclear cells. Adoptively-transferred EAE mice received weekly treatment of p40 (200 ng/mouse) via i.p. injection from 8 dpt. On 16 dpt, single-cell suspensions from cerebellum (A, C & E) and spinal cord (B, D & F) were stained with Zombie Aqua™ Fixable Viability Kit (Biolegend). Cells were washed with FACS buffer (Thermo-Scientific) and stained with CD4-FITC and CD8-APC-Cy7 (Biolegend) for extracellular stains. Cells were then stained IL-17-APC, and IFNγ-PE. Multicolor flow cytometric analyses were performed using LSRFortessa analyzer (BD Biosciences) and analyzed using FlowJo Software (v10) (A, cerebellum; B, spinal cord). The MFI of IFNγ (C, cerebellum; E, spinal cord), and IL-17 (D, cerebellum; F, spinal cord) in CD4$^+$ population was determined by using the CellQuest software. $^{***}p < 0.001$; $^{**}p < 0.01$; $^{*}p < 0.05$.

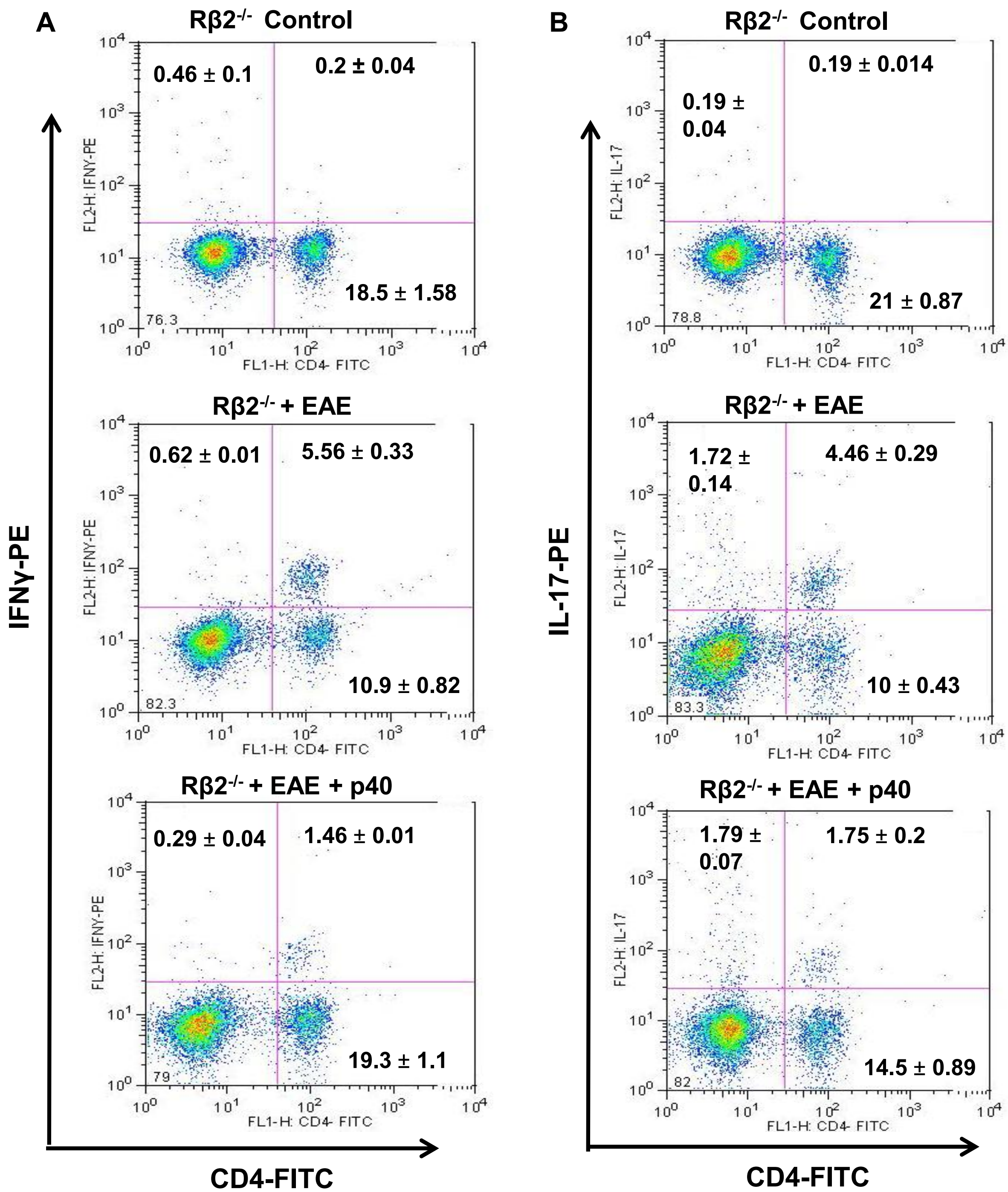

Figure S17. The p40 suppresses Th1 and Th17 responses *in vivo* in spleen of EAE in IL-12Rβ2$^{-/-}$ mice. EAE was induced in IL-12Rβ2$^{-/-}$ mice by MOG immunization. On 8 day post-immunization (dpi), mice received weekly treatment of p40 (200 ng/mouse) via i.p. injection followed by analysis of splenocytes by FACS in LSRFortessa analyzer (BD Biosciences) for CD4 & IFNγ (A) and CD4 & IL-17 (B) on 20 dpi. Results represent three independent experiments.

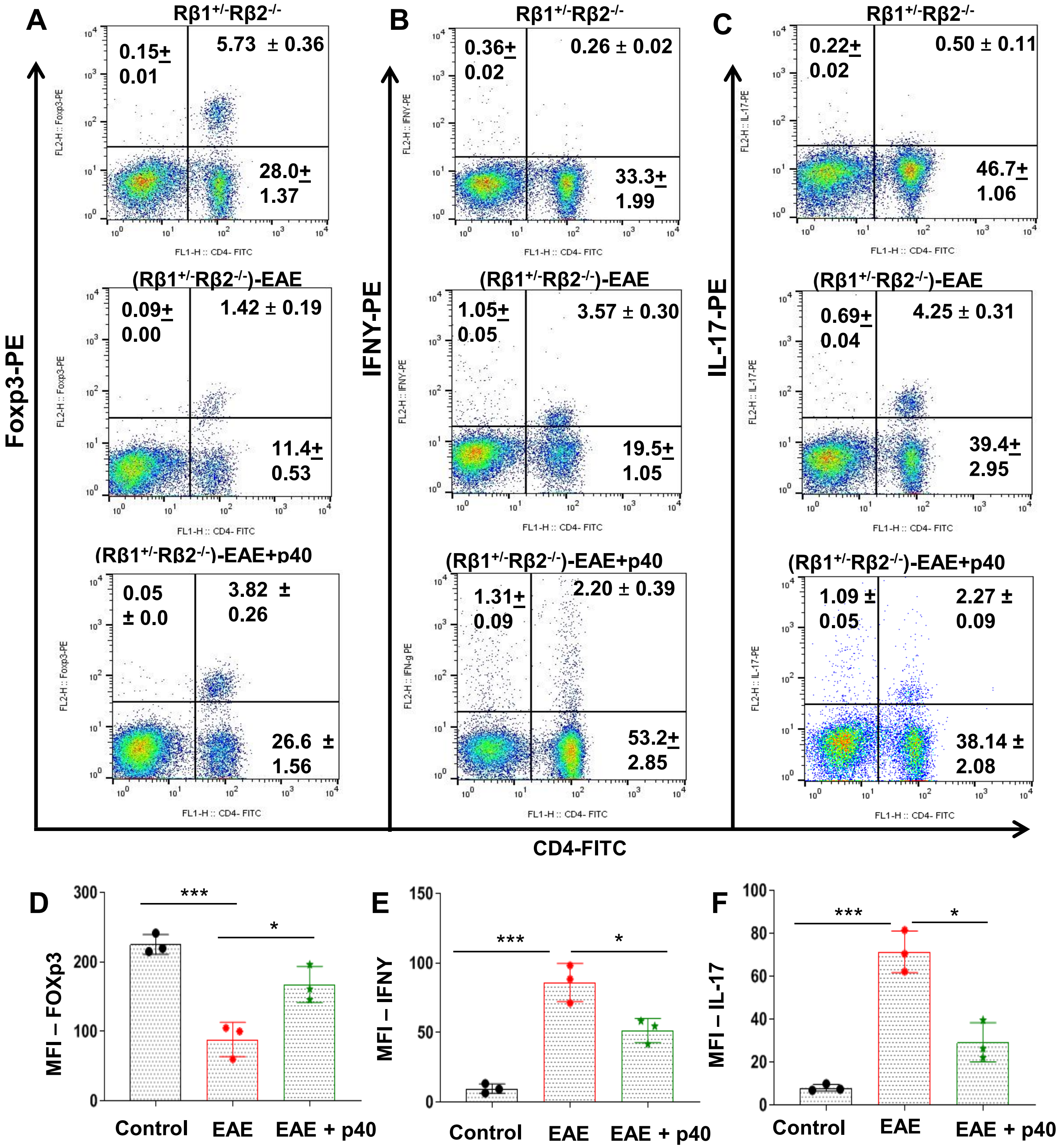

Figure S18. Effect of p40 on Th1, Th17 and Treg responses in MOG-induced active EAE in IL-12Rβ1$^{+/-}$IL-12Rβ2$^{-/-}$ (Rβ1$^{+/-}$Rβ2$^{-/-}$) mice. EAE was induced in Rβ1$^{+/-}$Rβ2$^{-/-}$ mice by MOG immunization. On 8 day post-immunization (dpi), mice received weekly treatment of p40 (200 ng/mouse) via i.p. injection followed by analysis of splenocytes by FACS in LSRFortessa analyzer (BD Biosciences) for CD4 & Foxp3 (A), CD4 & IFNγ (B) and CD4 & IL-17 (C) on 20 dpi. The MFI of Foxp3 (D), IFNγ (E) and IL-17 (F) in CD4^{+} population was calculated by using the CellQuest software. Data are mean ± SD of three different experiments. $^{*}p < 0.05$; $^{**}p < 0.01$; $^{***}p < 0.001$.

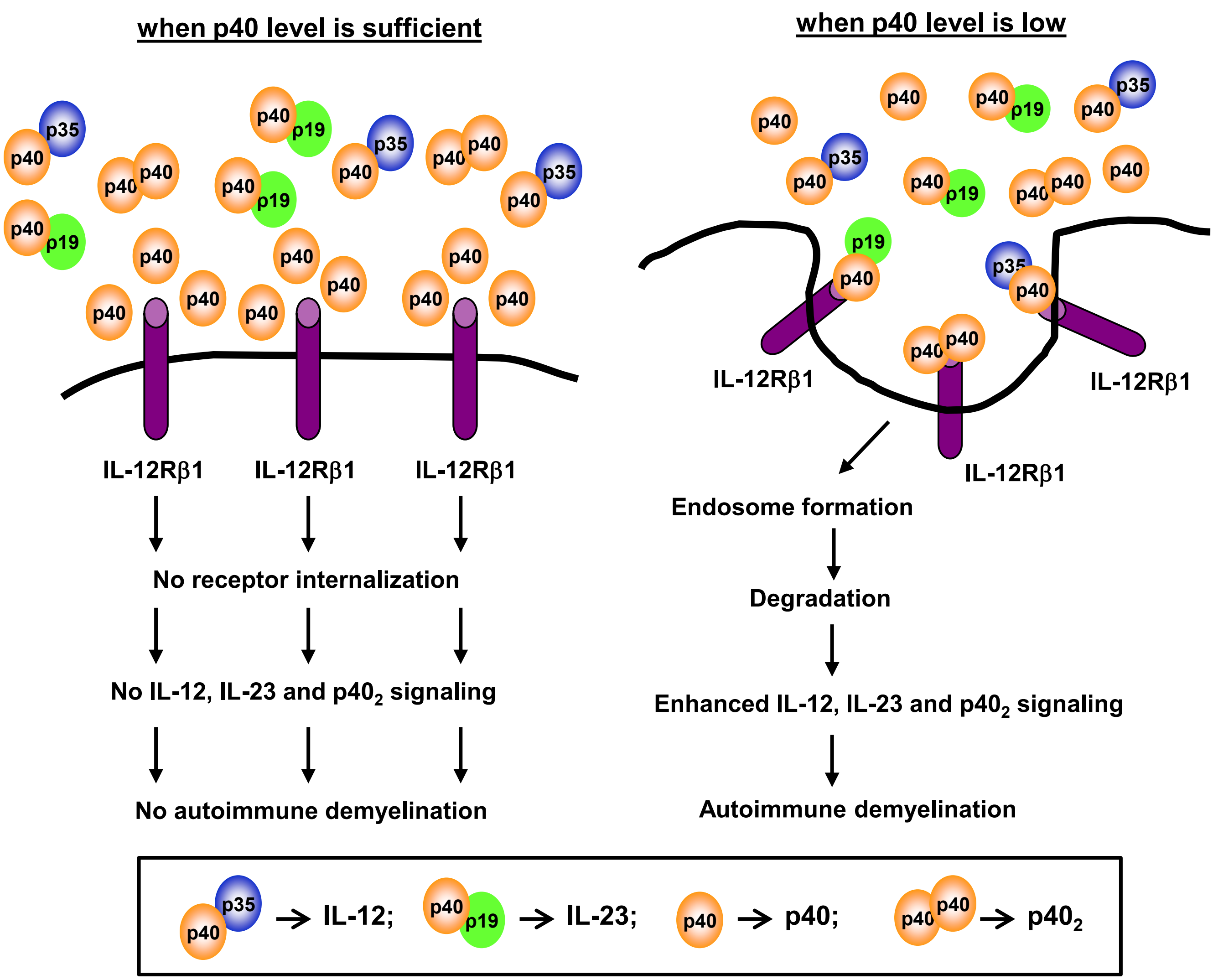

Figure S19. The schematic presentation of scientific summary. When more p40 is available as compared to IL-12 (p40:p35), IL-23 (p40:p19) and $p40_2$ (p40:p40), the internalization of IL-12Rβ1 is blocked (left side), leading to suppression of autoimmune demyelination. On the other hand, when there are more IL-12, IL-23 and $p40_2$, these molecules bind to IL-12Rβ1 strongly, cause its internalization and transduce signals for autoimmune pathology (right side).
